# LSM12 promotes the lung squamous cell carcinoma progression through mediating alternative splicing of ARRB1

**DOI:** 10.1038/s42003-025-08193-7

**Published:** 2025-05-27

**Authors:** Lin Wu, Fangyuan Zhang, Huanhuan Chen, Gang Zhao

**Affiliations:** 1 Department of Thoracic Surgery, Shengjing Hospital of China Medical University, Shenyang, China; 2 Department of General Surgery, Shengjing Hospital of China Medical University, Shenyang, China; 3 Department of Oncology, Shengjing Hospital of China Medical University, Shenyang, China; 4 Department of Radiology, Shengjing Hospital of China Medical University, Shenyang, China

**Keywords:** Lung cancer, Cell growth

## Abstract

Like-Smith protein 12 (LSM12), an RNA-binding protein, is highly expressed in tumor tissues of patients with lung squamous cell carcinoma (LUSC). However, the role of LSM12 in LUSC is unclear. In this study, overexpression of LSM12 promotes the proliferation, migration, and invasion and prevents the apoptosis of LUSC cells. In vivo, LSM12 accelerates the tumor growth and metastasis of LUSC cells using male BALB/c nude mice. Furthermore, we find that the Sterile alpha motif domain containing 4A (SAMD4A) is directly bound to the mRNA of LSM12 and accelerates the mRNA degradation. High-throughput omics analysis is performed to identify the potential target genes of LSM12 in LUSC cells. LSM12 regulates alternative splicing events and increases exon 13 skipped splicing of ARRB1 and mRNA expression. Our findings may provide fundamental research for the investigation of the development of LUSC and the potential role of LSM12 in LUSC cells.

## Abstract (web summary)

The molecular characterization of the activity of the RNA-binding protein LSM12 and its interplay with SAMD4A reveals that LSM12 promotes lung squamous cell carcinoma progression by regulating alternative splicing of the ARRB1 gene.

## Introduction

Nowadays, the leading cause of cancer-related death is still lung cancer^1^. About 85% of lung cancers are non-small cell lung cancer (NSCLC), which includes lung squamous cell carcinoma (LUSC), lung adenocarcinomas, and lung large cell carcinoma^2,3^. LUSC is the second most common histological subtype of LC, with approximately 30% of all cases^4^. Moreover, LUSC is regarded as an aggressive cancer. Typically, LUSC is only detected after metastasis has occurred^5^. The 5-year survival rate for LUSC is estimated to be less than 15%^6^. Because of the limited efficacy of LUSC treatment, exploring the underlying mechanisms actively is necessary for finding more targeted treatments.

Like-Smith protein 12 (LSM12) is a member of the LSM protein family. It complexes with nicotinic acid adenine dinucleotide phosphate (NAADP) to participate in activating the NAADP-evoked two-pore channel and mobilizing Ca^2+^ from acidic stores^7,8^. LSM12 is also an RNA-binding protein that interacts with RNAs of target genes and is critical for post-transcriptional events, including processing and transportation of RNA^9,10^. LSM12 forms a protein complex with other proteins and binds to the 3’-UTR (Untranslated Region) of the target mRNA to stabilize the mRNA in yeast^11^. Knockdown of LSM12 decreases EPAC1 expression via the 5’-UTR in human SH-SY5Y cells^12^. Alternative splicing (AS) events can generate multiple mRNAs from a single gene, which is important for gene expression^13^. Yan Dong et al. found that LSM12 functions as a splicing factor to regulate AS events of targeting RNA^14^. Studies found that LSM12 plays an important role in many cancers. Overexpression of LSM12 promotes the proliferation, migration, and invasion of oral squamous cell carcinoma (OSCC) cells. Moreover, the downregulation of LSM12 inhibits the proliferation and invasion of hepatocellular carcinoma cells^15^. In human neuroblastoma cells, lack of LSM12 promotes the injury of nucleocytoplasmic transport and nuclear integrity, which are vital for the normal cellular functions^12^. We found that LSM12 expression was significantly upregulated in LUSC tissues based on publicly available data and analysis of clinical samples. However, the role of LSM12 in LUSC remains unclear.

Sterile alpha motif domain containing 4A (SAMD4A) is a mammalian homolog of *Drosophila Smaug* and belongs to the SAMD4 family that mediates post-transcriptional regulation and translational repression in eukaryotes^16,17^. SAMD4A is an RNA-binding protein, and it can restrain the angiogenesis of breast tumors^18^. Additionally, knockdown of SAMD4A suppresses the proliferation, invasion, and migration of gastric cancer cells in vitro^19^. We found that SAMD4A expression was significantly downregulated in tumor tissues of patients with LUSC in the GEPIA-LUSC dataset.

In this study, we predicted the interaction between SAMD4A and RNA of LSM12 using catRAPID (http://s.tartaglialab.com/page/catrapid_group). It is unclear whether SAMD4A affects LUSC cells and regulates the LSM12 expression in LUSC cells. The present study explored that LSM12 is highly expressed in tumor tissues of LUSC patients and cancer progression by increasing cell proliferation, migration, and invasion, and decreasing the apoptosis of LUSC cells. SAMD4A inhibits LSM12 by accelerating the mRNA degradation. mRNA-seq and RIP-seq analysis demonstrated that LSM12 regulates alternative splicing events and increases exon 13 skipped splicing of ARRB1 and mRNA expression to promoting cancer progression of LUSC.

## Results

### LSM12 is upregulated in tumor tissues of LUSC based on public databases

**Fig. 1:**
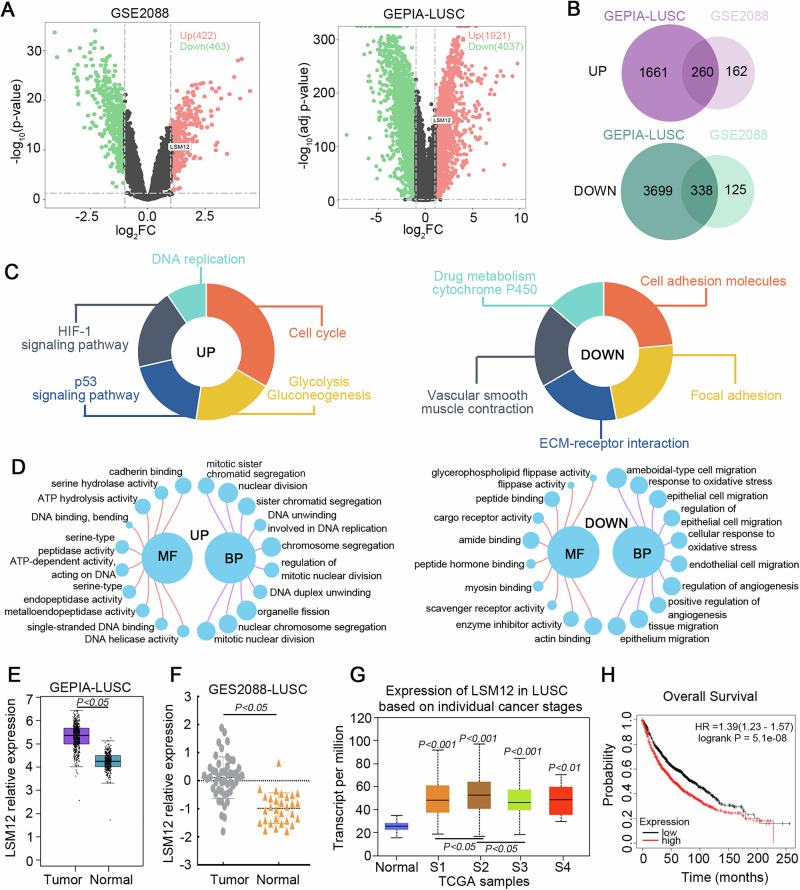
LSM12 is upregulated in tumor tissues of patients with LUSC. **A** The volcano plots of DEGs in the GEO dataset (GSE2088) and GEPIA-LUSC dataset. **B** The Venn diagrams depicted the overlapped upregulated DEGs and downregulated DEGs in the two datasets. **C** The main pathways that overlapped DEGs were enriched in KEGG. **D** The main terms in biological processes (BP) and molecular function (MF) for these DEGs enriching in GO terms. **E**, **F** The mRNA expression of *LSM12* in the GEPIA-LUSC dataset and the GSE2088-LUSC dataset. **G** The mRNA expression of *LSM12* in LUSC tissues based on individual cancer stages from TCGA samples (UALCAN database). **H** The relationship between LSM12 and overall survival of lung cancer.

Bioinformatics analysis on these two datasets showed the upregulated and downregulated differential expression genes (DEGs) in the GSE2088 and GEPIA-LUSC datasets (Fig. 1A). The Venn plots displayed that there were 260 overlapped upregulated DEGs and 338 downregulated DEGs in two datasets (Fig. 1B). In the KEGG analysis, these DEGs were enriched in the pathways that related to cancer development (Fig. 1C). Furthermore, GO analysis also showed that these overlapped DEGs were linked to the proliferation, migration, and division of cells (Fig. 1D). We found that LSM12 is upregulated in the tumor tissues of LUSC in comparison to normal tissues in two datasets (Fig. 1E, F). The expression levels of LSM12 are higher in individual cancer stages of LUSC than in normal tissues based on the UALCAN database (https://ualcan.path.uab.edu/index.html) (Fig. 1G). Lung cancer patients with high LSM12 expression have lower overall survival rates (Fig. 1H). Thus, the role of LSM12 in LUSC is further explored.

### LSM12 is upregulated in tumor tissues from LUSC patients and promotes the proliferation of LUSC cells

**Fig. 2:**
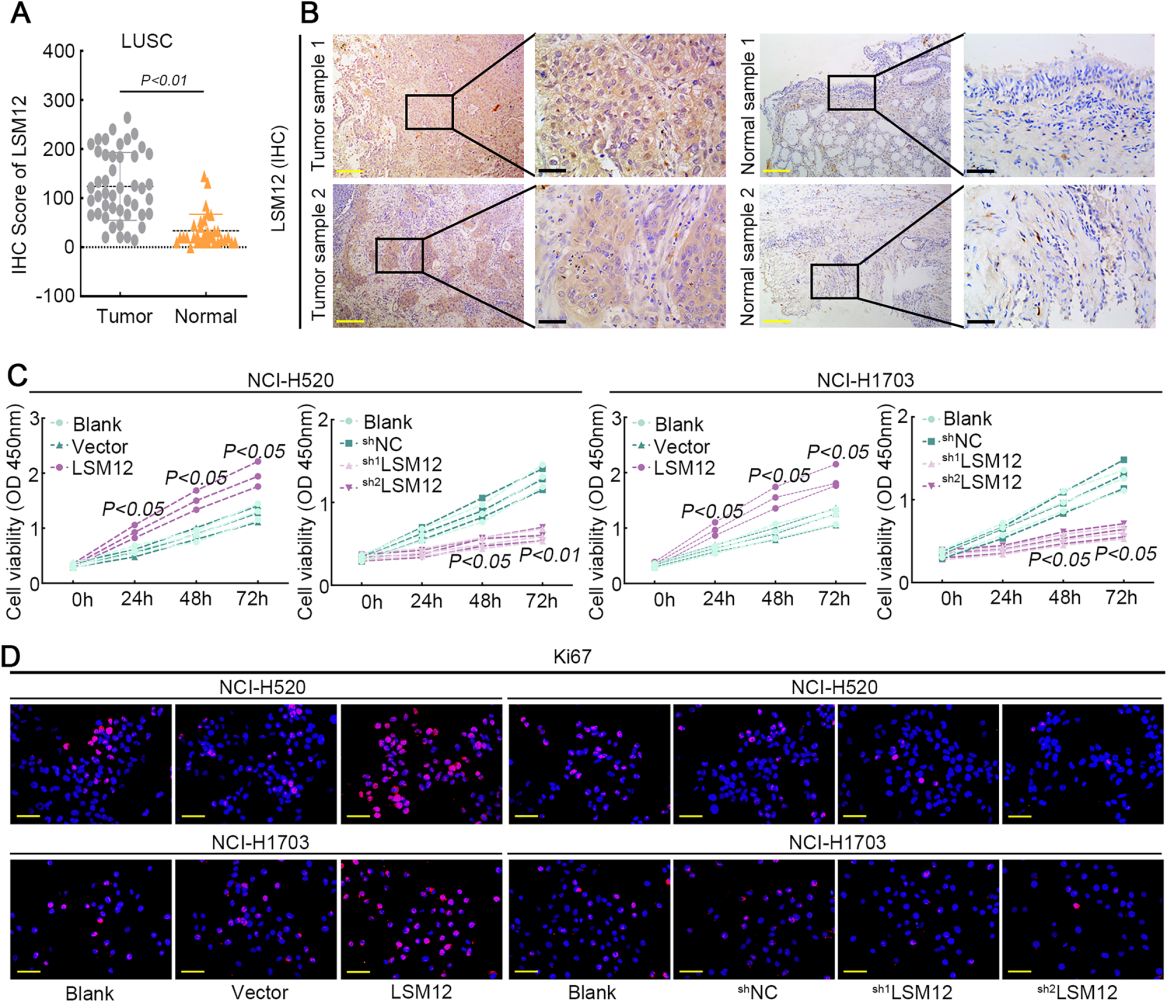
LSM12 promoted the proliferation of LUSC cells. **A** The IHC score of LSM12 expression levels by IHC staining in the tumor tissues (*N* = 46) and normal tissues (*N* = 33) from LUSC patients. **B** Representative images of IHC staining using LSM12 antibody in LUSC tissues and normal tissues. The yellow scale bar was 200 μm, and the blank scale bar was 50 μm. **C** The cell proliferation of LUSC cells at different times. **D** The expression of Ki67 was visualized with immunofluorescence staining. The scale bar was 50 μm. *N* = 3, and the data were presented as mean ± standard deviation (SD). Significant differences were indicated as *p* < 0.05.

**Fig. 3:**
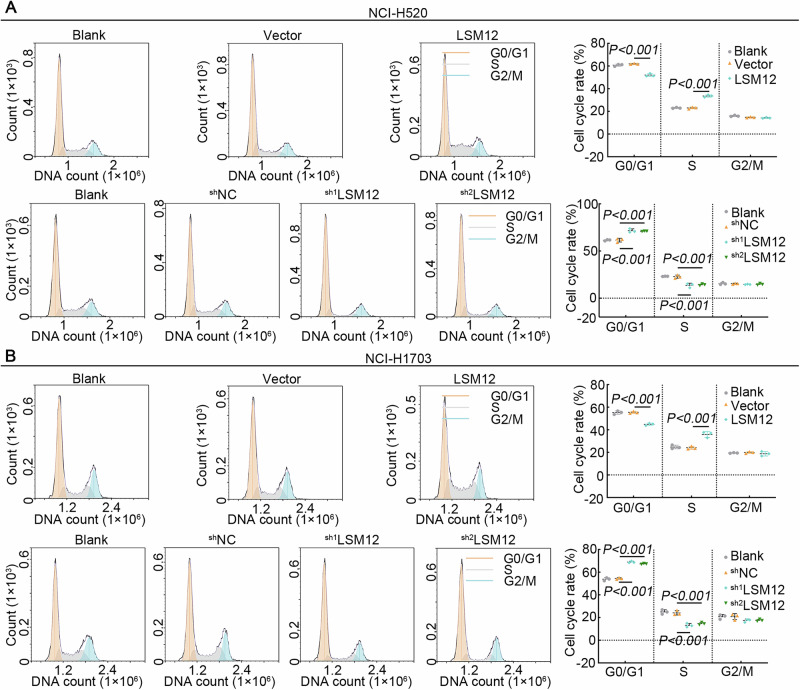
LSM12 promoted the cell cycle progression of LUSC cells. **A**, **B** The cell cycle distribution of LUSC cells was detected with flow cytometry. *N* = 3, and the data were presented as mean ± standard deviation (SD). Significant differences were indicated as *p* < 0.05.

The fixed paraffin tumor and normal tissues from the LUSC patients were used to detect the LSM12 expression determined by the immunohistochemical (IHC) assay. The H-score showed that LSM12 was upregulated in tumor tissues compared to normal tissues (Fig. 2A, B). Regarding the clinical correlation, high LSM12 expression was significantly associated with the T stage, TNM stage, and Tumor size in LUSC patients (Table 1). LUSC cell line NCI-H520 and NCI-H1703 cells were infected with lentivirus to overexpress or silence LSM12, and the LSM12 expression was observed using GFP immunofluorescence and real-time quantitative PCR (Real-time qPCR) (Supplementary Fig. 1A, B). In LUSC cells, overexpression of LSM12 enhanced cell proliferation, whereas down-regulation of LSM12 decreased cell proliferation, as determined by CCK-8 assay (Fig. 2C) and ki67 immunofluorescence staining (Fig. 2D). The cell cycle of LUSC cells was detected with Flow cytometry (FCM). The percentage of LUSC cells in the G0/G1 phase was decreased, and the percentage of cells in the S phase was increased following LSM12 overexpression. The changes in LUSC cell percentage in the G0/G1 phase and S phase displayed the opposite trend as that in the LSM12-silencing cells (Fig. 3A, B, and Supplementary Fig. 2). In summary, LSM12 promoted the proliferation of LUSC cells.

**Table 1:** Correlation between LSM12 expression level and clinicopathological features of LUSC patients

Characteristics	Cases	LSM12 expression level		*P* value
		Low	High	
Overall				
Gender				
Male	41	23	18	0.05812
Female	5	0	5	
Age				
≤60	15	9	6	0.34539
>60	31	14	17	
T stage				
T1-T2	37	23	14	0.00295
T3-T4	9	0	9	
N stage				
N0	33	14	19	0.10157
N1-N2	13	9	4	
TNM stage				
I-II	40	23	17	0.0286
III-IV	6	0	6	
Tumor size				
≤3 cm	27	17	10	0.03607
>3 cm	19	6	13	
Lymph node metastasis				
Yes	13	9	4	0.10157
No	33	14	19	

### LSM12 inhibits the apoptosis of LUSC cells

**Fig. 4:**
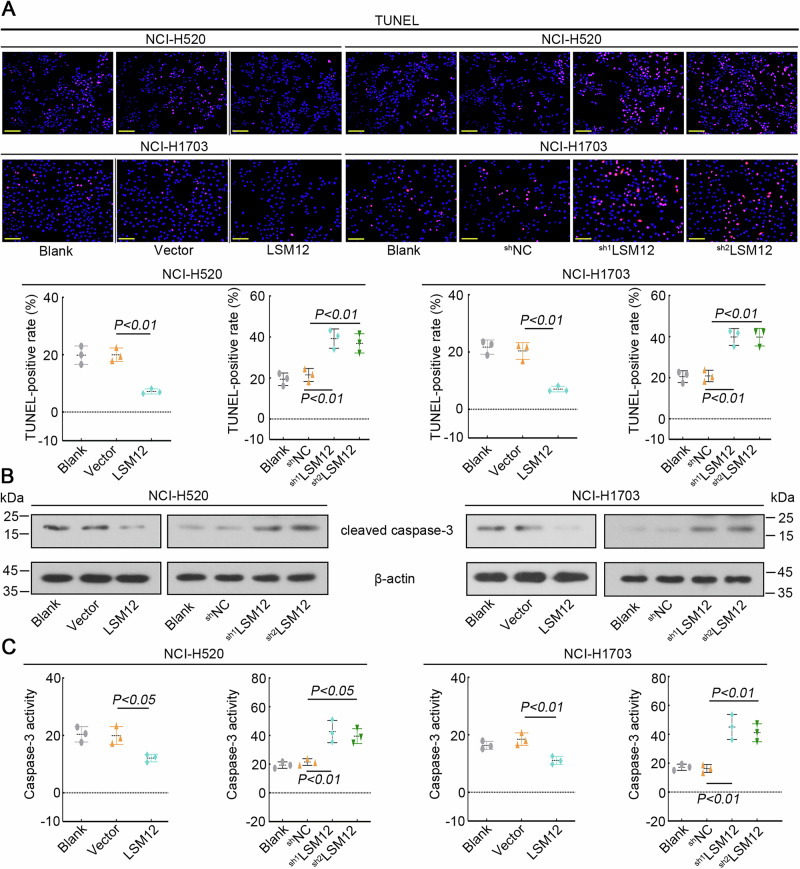
LSM12 inhibited the apoptosis of LUSC cells. **A** Apoptosis of LUSC cells was visualized with TUNEL staining. The scale bar was 100 μm. **B** The protein level of cleaved caspase-3 was measured. **C** The activity of caspase-3 was assessed with the commercial kit. *N* = 3, and the data were presented as mean ± standard deviation (SD). Significant differences were indicated as *p* < 0.05.

Knockdown LSM12 promoted the apoptosis of NCI-H520 and NCI-H1703 cells, while the apoptosis was restrained via overexpressing LSM12, as determined by TUNEL staining (Fig. 4A). The protein level of pro-apoptotic cleaved caspase-3 was decreased by overexpressing LSM12 and increased by silencing LSM12 in LUSC cells (Fig. 4B). Similarly, overexpression of LSM12 inhibited the activity of caspase-3, whereas down-regulation of LSM12 enhanced caspase-3 activity (Fig. 4C). The results indicated that LSM12 prevented the apoptosis of LUSC cells.

### LSM12 promotes the migration and invasion of LUSC cells

**Fig. 5:**
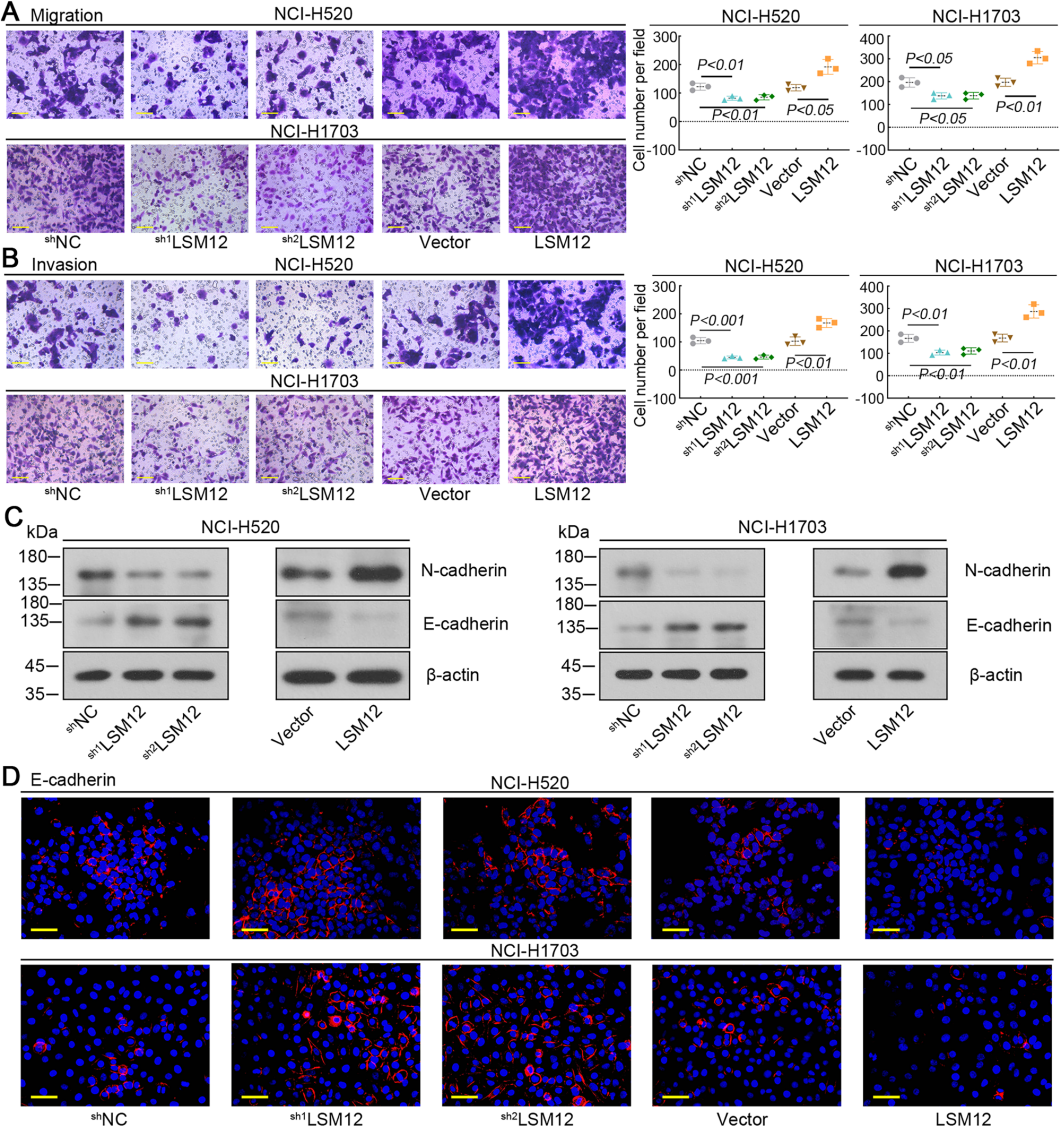
Migration and invasion of LUSC cells were enhanced by LSM12. Migration (**A**) and invasion (**B**) of LUSC cells were assessed with transwell assay. The scale bar was 100 μm. **C** The protein levels of N-cadherin and E-cadherin were detected. **D** The expression of E-cadherin was visualized with immunofluorescence staining. The scale bar was 50 μm. *N* = 3, and the data were presented as mean ± standard deviation (SD). Significant differences were indicated as *p* < 0.05.

Overexpression of LSM12 promoted the migration of NCI-H520 and NCI-H1703 cells, while silencing LSM12 prevented it (Fig. 5A). Similarly, the number of invasive cells increased in LSM12 overexpressing cells, whereas the number of invasive cells decreased upon down-regulation of LSM12 (Fig. 5B). Additionally, the expression of N-cadherin was increased through overexpressing LSM12, but E-cadherin expression was decreased. It indicated that LSM12 overexpression promoted the epithelial-mesenchymal transition (EMT) of LUSC cells. Knockdown of LSM12 inhibited the EMT via downregulating N-cadherin and upregulating E-cadherin (Fig. 5C, D). The results suggested that LSM12 promotes the migration and invasion of LUSC cells.

### LSM12 promotes tumor growth and metastasis of LUSC cells in nude mice

**Fig. 6:**
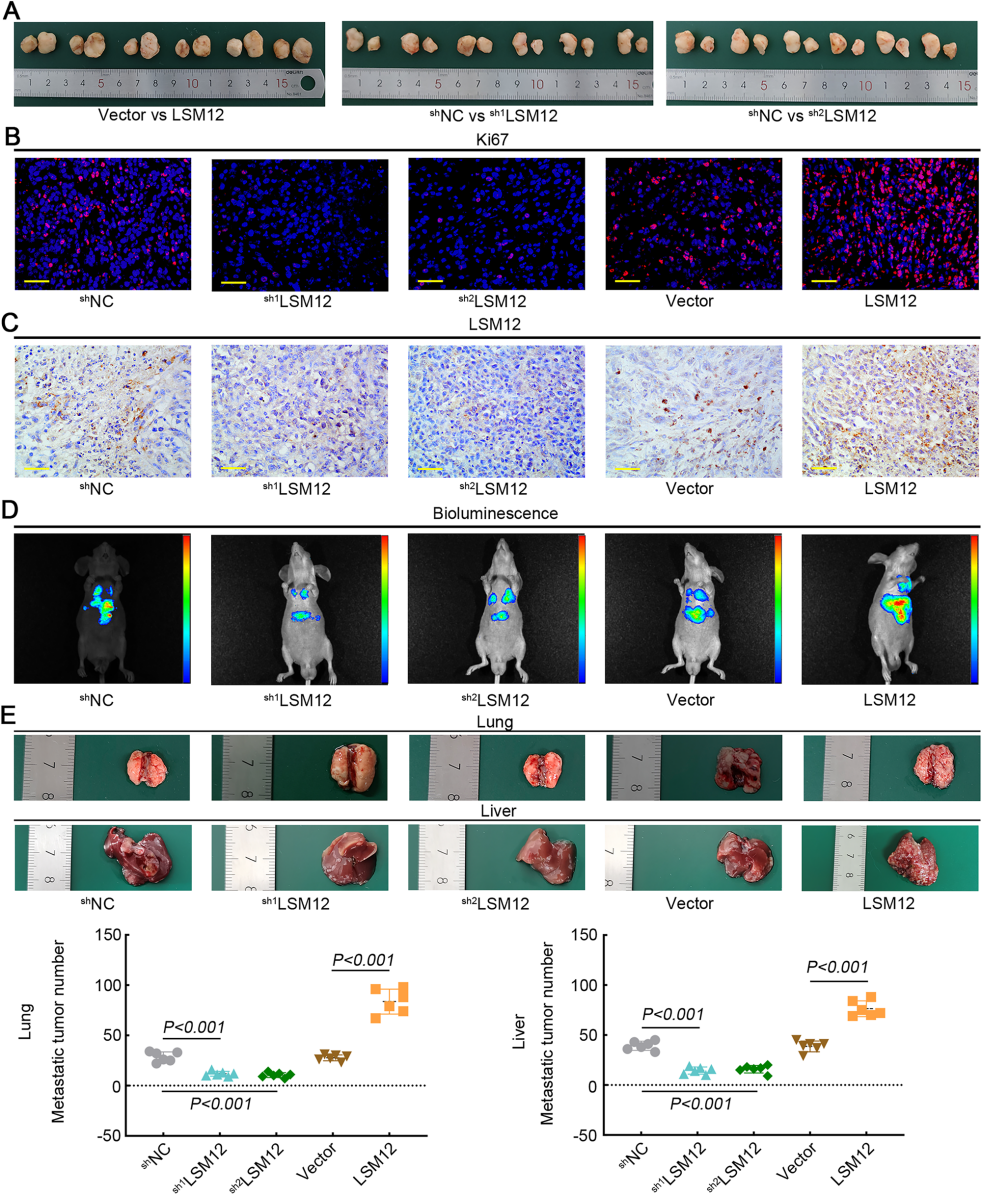
LSM12 promoted tumor growth and metastasis of LUSC cells in mice. **A** Images of transplanted tumors that were harvested from different groups. **B** The expression of Ki67 was visualized with immunofluorescence staining. The scale bar was 50 μm. **C** The expression of LSM12 was visualized with immunohistochemistry staining. The scale bar was 50 μm. **D** Bioluminescence imaging of nude mice that were injected with LUSC cells. Fluorescence intensity is increased from the blue to red. **E** Images of lung and liver tissues, which were harvested from nude mice. The number of metastatic nodules in lung and liver tissues in mice. *N* = 6, and the data were presented as mean ± standard deviation (SD). Significant differences were indicated as *p* < 0.05.

Overexpression of LSM12 in NCI-H1703 cells promoted the growth of transplanted tumors in nude mice, and knockdown of LSM12 prevented the tumor growth (Fig. 6A). In Fig. 6B, the cell proliferation marker Ki67 was decreased in the mice injected with LSM12-silenced NCI-H1703 cells and increased in the mice that received LSM12-overexpressing cells. The expression of LSM12 was confirmed by IHC staining in Fig. 6C. To further explore the role of LSM12 in tumor metastasis, nude mice were injected with LUSC cells through the tail vein. The in vivo imaging system exhibited that nude mice had higher bioluminescence in the lung and liver of mice injected with LSM12-overexpressed NCI-H1703 cells than control mice, and lower fluorescence intensity in mice received with LSM12-silenced cells (Fig. 6D). The number of metastatic nodules in the lung and liver increased with LSM12 overexpression, while silencing LSM12 in mice caused the opposite changes (Fig. 6E). These results supported that LSM12 promoted the tumor growth and metastasis of LUSC cells in vivo.

### SAMD4A overexpression prevented the malignant phenotype of LUSC cells by regulating the stability of *LSM12* mRNA

**Fig. 7:**
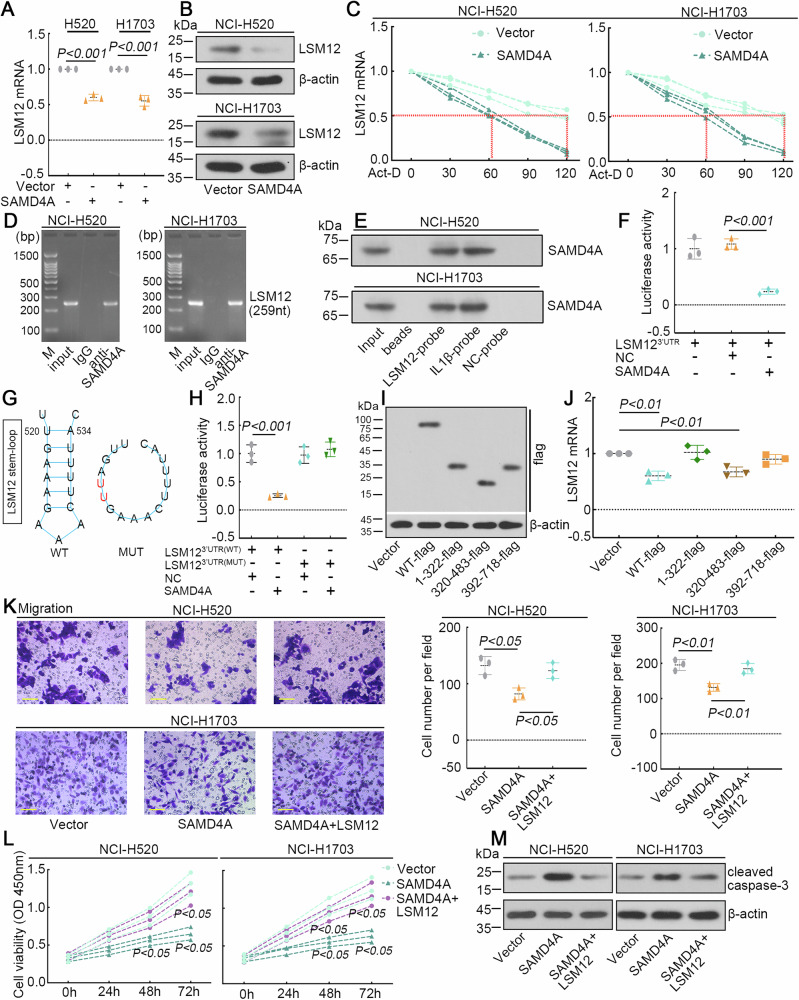
SAMD4A restrained the malignant phenotype of LUSC cells by regulating the LSM12 mRNA stability. **A**, **B** The mRNA and protein expressions of LSM12 in LUSC cells after SAMD4A overexpression. **C** The mRNA level of *LSM12* at different times after Act-D treatment. **D**, **E** The interaction between *SAMD4A* and *LSM12* mRNA was detected with RIP-PCR and RNA pull-down (IL1β-probe beads as a positive target and scrambled probe-beads as a negative control for SAMD4A). **F** HEK293T cells were co-transfected with the pmirGLO luciferase reporter inserting the sequence of LSM12 including the 3’UTR region, and the SAMD4A overexpressed plasmid. The luciferase activities were measured. **G** Schematic diagram of the predicted conserved stem-loop structure of LSM12 wild-type (WT) 3’ UTR and non-stem loop structure of mutant (MUT) 3’ UTR through replacing 2 nucleotides (red character). **H** The pmirGLO luciferase reporter containing WT 3’ UTR or MUT 3’ UTR was co-transfected with SAMD4A. The luciferase activities were measured. **I** Expressions of SAMD4A truncated fragments were confirmed by western blot. **J** The mRNA level of *LSM12* was measured by real-time qPCR after overexpressing SAMD4A and its fragments in NCI-H1703 cells. **K** Migration of LUSC cells was assessed by transwell assay. The scale bar was 100 μm. **L** The cell proliferation of LUSC cells was measured. **M** The expression of cleaved caspase-3 was detected by western blot. *N* = 3, and the data were presented as mean ± standard deviation (SD). Significant differences were indicated as *p* < 0.05.

The mRNA expression of *LSM12* is negatively related to the mRNA expression of *SAMD4A* in the tissues combined with LUSC tumors and normal lung tissues based on the GEPIA database (Supplementary Fig. 3A). SAMD4A is downregulated in the tumor tissues of LUSC in comparison to normal tissue (Supplementary Fig. 3B). We predicted that SAMD4A can interact with *LSM12* mRNA by the catRAPID database. Therefore, whether SAMD4A can regulate the expression of LSM12 was determined. First, NCI-H520 and NCI-H1703 cells were infected with lentivirus to overexpress SAMD4A-GFP fusion protein efficiently (Supplementary Fig. 3C, D). Overexpression of SAMD4A reduced the mRNA level and protein level of LSM12 in NCI-H520 and NCI-H1703 cells (Fig. 7A, B). Overexpression of SAMD4A accelerated the decrease of *LSM12* mRNA levels in NCI-H520 and NCI-H1703 cells (Fig. 7C). RIP-PCR and RNA pull-down assay verified that SAMD4A interacts with the mRNA of *LSM12* in LUSC cells (Fig. 7D, E). The detection of IL1β and THBS1 in the RIP-PCR assay was considered, respectively, as a positive target control and a negative control for SAMD4A (Supplementary Fig. 3E). HEK293T cells were co-transfected with the SAMD4A overexpressed plasmid and a pmirGLO luciferase reporter inserting the 3’UTR region of LSM12. SAMD4A suppressed luciferase activities of luciferase reporter, suggesting that SAMD4A targeted the 3’UTR of LSM12 (Fig. 7F). Zhou et al. found that SAMD4A binds to the conserved stem-loop structure in the 3’UTR of target transcripts^18^. Comparing the 3’UTR sequences of LSM12 from different species and generating the secondary structure of the RNA using the ViennaRNA Web Services (http://rna.tbi.univie.ac.at/), we found that the stem-loop formed at the 520–534 of LSM12 3’UTR (homo sapiens) is conserved across multiple species (Supplementary Fig. 3F). By replacing two nucleotides at 523–524 of LSM12 3’UTR, the LSM12 3’UTR formed a non-stem loop structure at the 520–534 (Fig. 7G). To verify whether SAMD4A binds to the conserved sequence that forms a stem-loop structure and affects the stabilization of *LSM12* mRNA, we used a luciferase reporter containing LSM12 wild-type (WT) 3’UTR and mutant (MUT) 3’UTR by replacing two nucleotides. The luciferase assay results indicated that the MUT 3’UTR eliminated the inhibitory effect of SAMD4A on *LSM12* mRNA (Fig. 7H). It indicated that SAMD4A binds to stem-loop structure containing the 520–534 of LSM12 3’UTR. To identify which domain of SAMD4A is required for downregulating *LSM12* mRNA, three different-length fragments (amino acid (aa) 1-322, aa320-483, and aa392-718) of SAMD4A were prepared. In Fig. 7I, the expressions of these fragments are detected. Only wild-type (WT) and fragment aa320-483 reduced the mRNA expression level of *LSM12* significantly (Fig. 7J).

Next, LUSC cells were coinfected with the SAMD4A-GFP and LSM12-GFP overexpressing lentivirus. The mRNA levels LSM12 were detected in Supplementary Fig. 3G. Overexpression of LSM12 didn’t affect the protein levels of SAMD4A (Supplementary Fig. 3H). We found that overexpression of SAMD4A reduced the migratory and proliferative capacities of LUSC cells, while overexpression of LSM12 rescued this decline (Fig. 7K, L). In addition, the expression of cleaved caspase-3 was increased by SAMD4A overexpression and downregulated by overexpressing LSM12 (Fig. 7M). It supported that SAMD4A promoted the apoptosis of LUSC cells, and overexpression of LSM12 restrained this process. These results indicated that SAMD4A acted as a tumor suppressor in LUSC by regulating the expression of LSM12.

### Identification of potential targets of LSM12 in LUSC cells

**Fig. 8:**
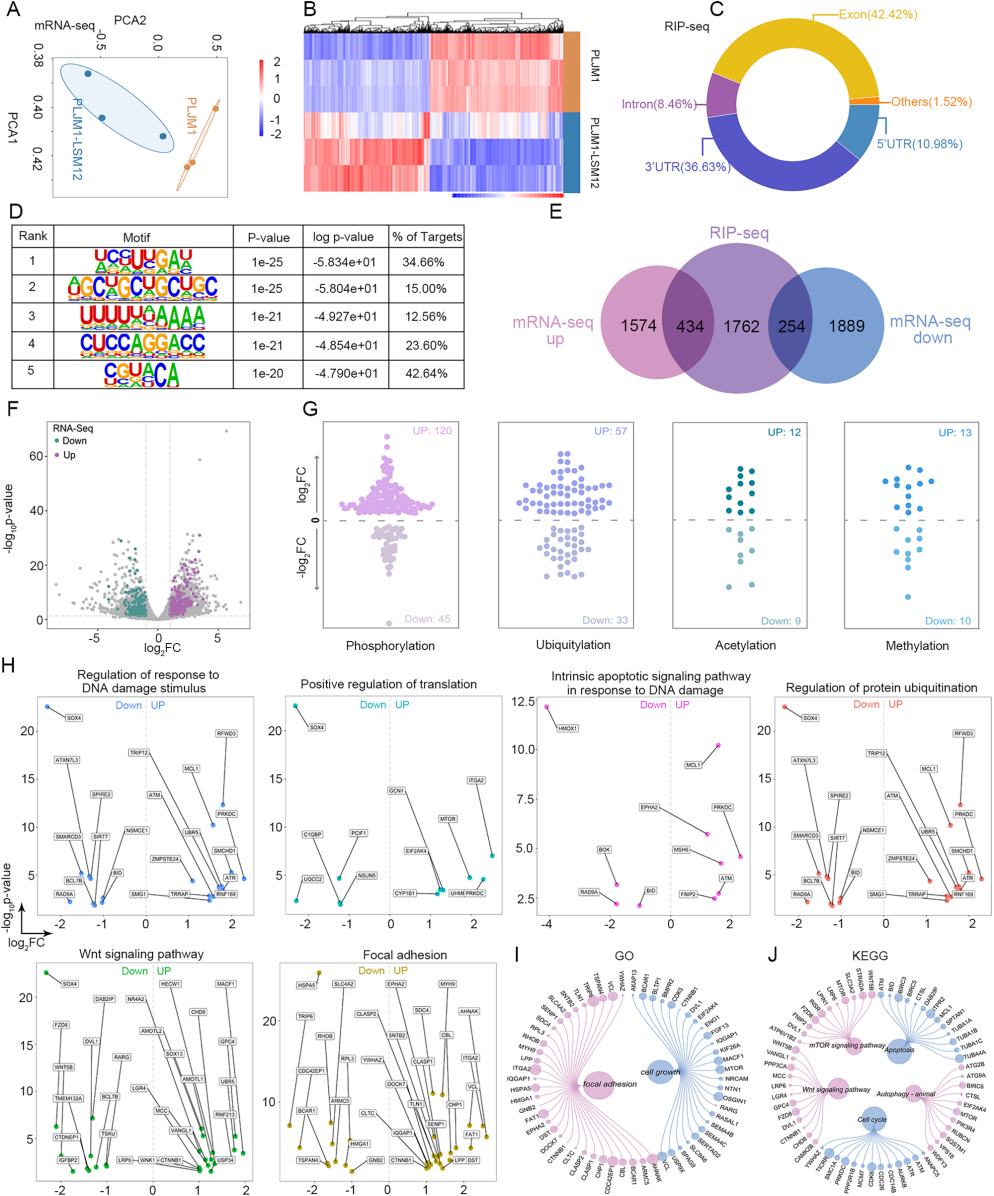
The identification of potential targets of LSM12 using mRNA-seq and RIP-seq. **A** Principal component analysis (PCA) of control (*N* = 3) and LSM12-overexpressing NCI-H1703 cells (*N* = 3) for mRNA-seq. **B** Heatmap of differentially expressed genes (DEGs) from RNA-seq analysis. **C** Ring chart of the distribution of LSM12 binding peaks based on RIP-seq data from NCI-H1703 cells. **D** Top five motifs of LSM12-binding sites detected by HOMER motif analysis from the RIP-Seq. **E** The Venn diagrams depicted the overlapping of the targets of LSM12 identified by RIP-seq and mRNA-seq analysis. **F** Volcano plot of mRNA-seq data in NCI-H1703 cells. The up- and down-regulated genes identified from RIP-seq are highlighted. **G** The annotated functions of overlapped genes related to phosphorylation, ubiquitination, acetylation, and methylation were summarized. **H** The GO enrichment pathways of overlapped upregulated and downregulated genes are related to cell proliferation, migration, and protein function. **I, J** The overlapped DEGs are related to cell growth, migration, apoptosis, and canonical signaling pathways from GO and KEGG enrichment analysis. The size of each dot is determined by the |log2FC| of each gene.

The potential targets of LSM12 in LUSC remain unclear. Finding out the downstream targets of LSM12 helped clarify the mechanism of LSM12 in LUSC cells. The mRNA-seq on NCI-H1703 cells with or without LSM12 overexpression and LSM12 RIP-seq were performed to identify the potential targets of LSM12. PCA of control and LSM12-overexpressing NCI-H1703 cells and the heatmap displayed the unsupervised hierarchical clustering of DEGs in six samples for mRNA-seq (Fig. 8A, B, and Supplementary data 1). Figure 8C showed that most of the LSM12 binding peaks mapped to Exon, 3’UTR, and 5’UTR regions. The top five motifs of LSM12-binding sites were detected by HOMER motif analysis from the RIP-Seq, as illustrated in Fig. 8D and Supplementary data 2. The overlapped genes were obtained from DEGs in mRNA-seq and genes in RIP-seq. There were 434 upregulated genes and 254 downregulated genes in both datasets (Fig. 8E, F). These overlapped genes are related to phosphorylation, ubiquitylation, acetylation, and methylation modification (Fig. 8G). GO enrichment pathways exhibited that many upregulated and downregulated genes are related to cell proliferation, migration, apoptosis, and protein function, respectively (Fig. 8H). Additionally, GO and KEGG enrichment pathways of total DEGs both in mRNA-seq and RIP-seq were also related to cell growth, migration, apoptosis, and canonical signaling pathways (Fig. 8I, J).

### LSM12 regulates the proliferation and migration of LUSC cells via mediating alternative splicing of ARRB1 RNA

**Fig. 9:**
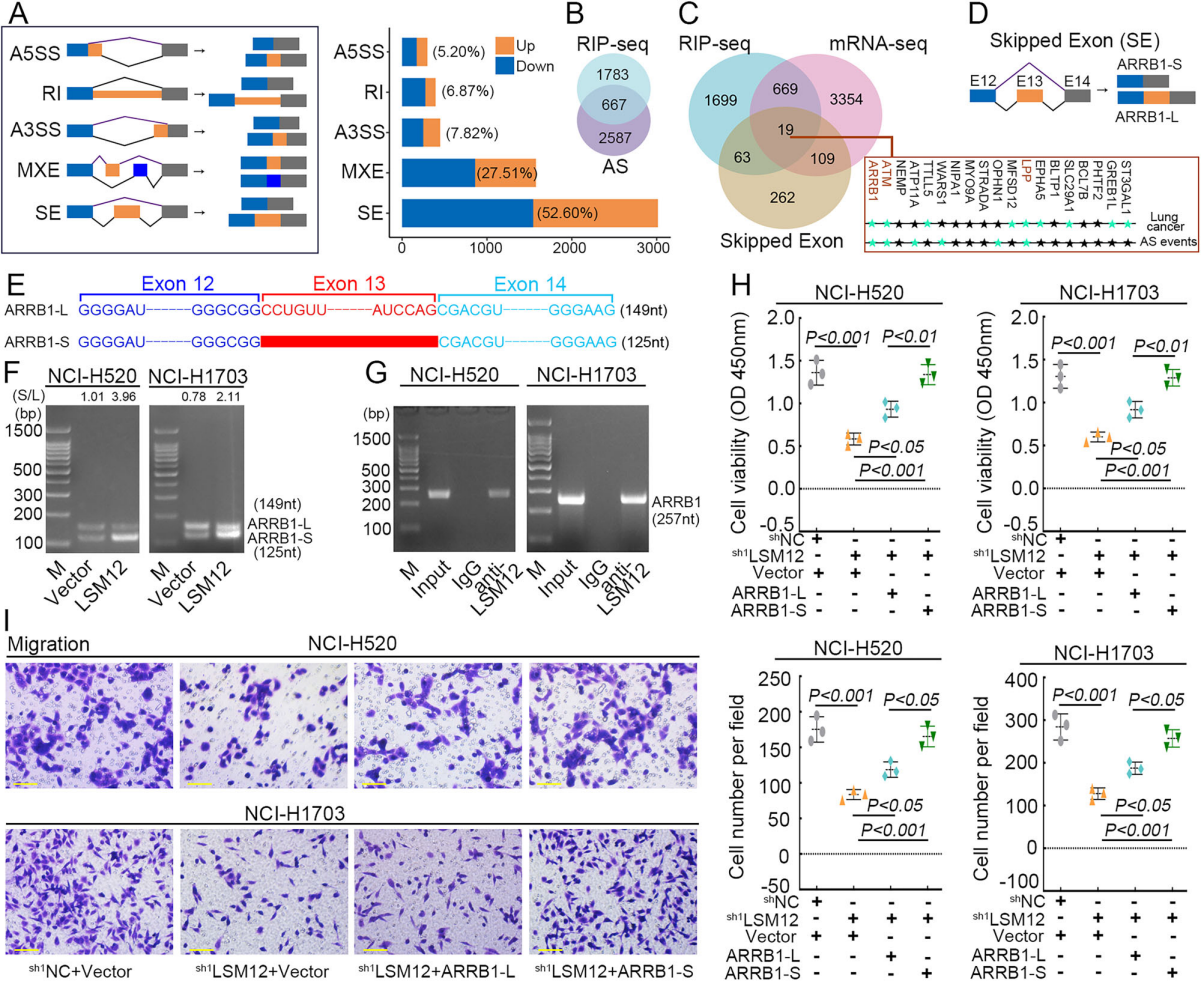
LSM12 regulates the proliferation and migration of LUSC cells via mediating alternative splicing of ARRB1 RNA. **A** Schematic diagram of five main types of alternative splicing (AS) events and differential AS analysis regulated by LSM12 from mRNA-seq data. **B** The Venn diagrams depicted overlapped genes from RIP-seq and AS events including A5SS, RI, A3SS, MXE, and SE. **C** The Venn diagrams depicted overlapped genes from RIP-seq, mRNA-seq, and Skipped Exon events. **D** Schematic diagram of the Skipping Exon of ARRB1. **E** Schematic diagram of the fragment of *ARRB1* mRNA containing Exon 13 (ARRB1-L) or skipping Exon 13 (ARRB1-S). **F** RT-PCR products for analysis of the ARRB1 isoforms (ARRB1-L and ARRB1-S) in LUSC cells. **G** The interaction between LSM12 and ARRB1 RNA was detected with RIP-PCR. **H, I** Cells were transfected ARRB1-L and ARRB1-S overexpressing vector into LSM12-silencing LUSC cells. Cell proliferation and migration were measured. SE, Skipped Exon, MXE, mutually exclusive exons, A3SS, alternative 3’ splice sites, A5SS, alternative 5’ splice sites, RI, retained intron. *N* = 3, and the data were presented as mean ± standard deviation (SD). Significant differences were indicated as *p* < 0.05.

In this study, we paid more attention to the role of LSM12 in regulating alternative splicing (AS). AS event analysis using mRNA-seq data showed thousands of LSM12-mediated differential AS events, including SE, MXE, A3SS, A5SS, and RI (Supplementary data 3). SE was the main type of AS event, accounting for 52.60% of the total AS events by LSM12 overexpression (Fig. 9A). There were 667 overlapping genes between RIP-Seq and the AS events (Fig. 9B). To screen the genes for subsequent studies, we overlapped the genes form the DEGs for mRNA-seq, the genes with higher differential SE events (|IncLevelDifference|> 0.3, *p*-value < 0.05), and the genes for RIP-seq. According to literature search on PubMed “https://pubmed.ncbi.nlm.nih.gov/”, among the 19 overlapping genes, only ARRB1 (Arrestin beta 1), ATM (ATM serine/threonine kinase) and LPP (LIM domain containing preferred translocation partner in lipoma) were reported to play a role in lung cancer and are mediated by AS events (Fig. 9C). ARRB1 expression is increased in tumor tissues of lung cancer including LUSC, and overexpression ARRB1 promotes cell proliferation^20,21^. Previous studies have reported that ARRB1 can undergo exon 13 jumping from long ARRB1 isoform (ARRB1-L) to short ARRB1 isoform (ARRB1-S, lacking exon 13) (Fig. 9D) and that ARRB1-S has a higher migratory capacity in human umbilical cord endothelial cells than ARRB1-L^22,23^. AS analysis and RT-PCR products revealed an increase in the expression of ARRB1-S and the ratio of ARRB1-S and ARRB1-L after overexpression of LSM12 (Fig. 9E, F). RIP-PCR assay verified that LSM12 binds with the RNA of ARRB1 (Fig. 9G). The results indicated that LSM12 regulates the alternative splicing of ARRB1 in LUSC cells. Next, ARRB1-L and ARRB1-S were transfected into LSM12-silencing LUSC cells. Overexpression of ARRB1-L and ARRB1-S can both promote the cell proliferation and migration of LUSC cells. More importantly, ARRB1-S had a more pronounced effect than ARRB1-L (Fig. 9H, I). The data suggest that LSM12 increases exon 13 skipped splicing of ARRB1 and mRNA expression to promote LUSC progress.

## Discussion

LUSC is one of the main subtypes of lung cancer. Because of the rapid progression of LUSC, most patients are diagnosed with a late stage, and the prognosis is poor^24^. In recent years, with the emergence of targeted therapies, many biomarkers, like specific genes, have been studied and used in the treatment of cancers^25^. Thus, clarifying the molecular mechanism of LUSC helps find its biomarkers and treat LUSC with targeted therapies. The LSM family plays an important role in the cancer process. For example, LSM2 is upregulated in the tumor tissues of patients with cutaneous skin melanoma, and knockdown of LSM2 reduces the cell proliferation and migration of cutaneous skin melanoma cells^26^. LSM4 is highly expressed in hepatocellular carcinoma tissues, and knockdown of LSM4 reduces the cell proliferation and migration of hepatocellular carcinoma cells^27^. LSM5 and LSM8 are potential biomarkers for gastric cancer^28^. LSM12 has been regarded as an oncogene in many cancers^29^. In colorectal cancer (CRC), LSM12 is highly expressed and participates in the proliferation, invasion, and apoptosis of CRC cells through the WNT/CTNNB1 pathway^30^. Additionally, LSM12 expression is upregulated in clinical tissues of OSCC, and knockdown of LSM12 significantly inhibited cell proliferation, migration, and invasion of OSCC cells in vitro and inhibited tumor formation in vivo^14^. In this study, we found that LSM12 was upregulated in the tumor tissues of patients with LUSC. Overexpression of LSM12 enhanced the proliferation, migration, and invasion and suppressed the apoptosis of LUSC cells in vitro. Moreover, overexpression of LSM12 promoted tumor growth and lung and liver metastasis in vivo.

We found that SAMD4A is upstream of LSM12 and a negative regulator of LSM12. In gastric cancer, SAMD4A plays a vital role and serves as a negative prognostic marker. Knockdown of SAMD4A inhibited cell proliferation, invasion, and migration abilities of gastric cancer cells^19^. However, SAMD4A is upregulated in breast tumor tissues, and overexpression of SAMD4A inhibited tumor growth and lung metastasis of breast cancer cells in vivo^18^. In this study, overexpression of SAMD4A inhibited cell proliferation and migration and increased cleaved caspase-3 levels in LUSC cells. Notably, overexpression of SAMD4A reduced the mRNA expression of *LSM12* in LUSC cells. RIP-PCR and RNA pull-down assay verified that SAMD4A, as an RNA-binding protein, was able to bind with *LSM12* mRNA. SAMD4A is an important paralog of SAMD4B^16^. SAMD4B inhibits the transcriptional activity of activator protein-1, p53, and p21, and the sterile alpha motif (SAM) is the main region of transcription suppression^31^. Similar to SAMD4B, the SAM domain of SAMD4A was the main function domain for transcription suppression, and it was able to restrain the stability of *LSM12* mRNA. Further study found that the suppressed malignant behaviors of LUSC cells in SAMD4A-overexpressing cells could be reversed by LSM12. The results showed that SAMD4A acted as a tumor suppressor in LUSC by decreasing the expression of LSM12.

The mRNA-seq data showed that overexpression of LSM12 affected the thousands of gene expressions in NCI-H1703 cells. LSM12, as an RNA-binding protein, has a similar mechanism of action as other members of the LSM family. It binds with the RNAs of targets and regulates the RNA stability. Thus, in this study, RIP-seq was used to reveal the binding downstream targets of LSM12. RIP-seq data showed that LSM12 mainly binds to the Exon, 3’UTR, and 5’UTR regions of RNAs. It is well-known that the 3’UTR region of mRNA is important for mRNA processes such as mRNA stability^32^. LSM12 has been reported to promote mRNA stability by binding 3’UTR and 5’UTR of targets. In addition, CDS binding with RNA-binding proteins can also regulate mRNA stability^33^. In this study, a total of 688 genes identified in RIP-seq overlapped with the DEGs from mRNA-seq in LUSC cells. It indicates that LSM12 may regulate the mRNA levels of these 688 genes by binding their mRNA and likely through different regulatory mechanisms. GO and KEGG analyses showed that many genes are related to regulating phosphorylation, ubiquitylation, acetylation, and methylation modification of their targets. Many of the genes also might participate in the important pathways of cell proliferation, migration, and apoptosis, which are crucial for cancer development. For instance, focal adhesion signaling and the Wnt signaling pathway are important for cell survival and the migration of cancer cells^34,35^. In addition, the pathways including the regulation of response to DNA damage stimulus and intrinsic apoptotic signaling pathway in response to DNA damage, are more likely related to the apoptosis of cells.

AS analysis using the mRNA-seq dataset revealed that LSM12 also affects the AS events in LUSC cells. Alternative splicing can regulate mRNA expression and isoform switching or both of them. For instance, YBX1 regulates MKNK1 splicing and affects the mRNA expression^36^. Splicing factor SRSF1 affects the isoform switching of many genes in breast cancer cells^37^. Additionally, SNORD14E recruits SRSF1 to promote FOXM1 skipped exon events, determined by increasing the FOXM1b and FOXM1c but not affecting FOXM1a. Overexpression of SNORD14E increased the mRNA stability of FOXM1. It indicates that SNORD14E doesn’t switch isoforms but promotes particular isoform expression^38^. Our results showed that LSM12 promoted ARRB1-S expression but didn’t observably affect ARRB1-L expression. The limitation of our study is that mRNA-seq and RT-PCR results indicated that LSM12 promoted the mRNA expression of *ARRB1*, but our results could not exclude the possibility that LSM12 promotes the total mRNA through other binding sites. More experiments will need to be performed in the future.

In summary, LSM12 was upregulated in LUSC cells and promoted cell proliferation, migration, and invasion of LUSC cells in vitro and enhanced tumor formation and metastasis in vivo. Additionally, SAMD4A affects the mRNA stability of *LSM12*, and overexpression of LSM12 reversed the effect of SAMD4A on LUSC cells. Moreover, many potential target genes of LSM12 were identified in LUSC cells, and ARRB1 was proven to be downstream of LSM12. In conclusion, our results provided the fundamental research for the investigation of the development of LUSC and the role of LSM12 in LUSC cells.

## Methods

### Bioinformatic analysis of GSE2088 dataset and GEPIA-LUSC dataset

The bioinformatic analysis of DEGs between tumor tissues of patients with LUSC and normal lung tissues from the GSE2088 dataset downloaded from the Gene Expression Omnibus database (GEO, https://www.ncbi.nlm.nih.gov/gds/) and GEPIA database (GEPIA-LUSC dataset) (http://gepia.cancer-pku.cn/) was performed. The 48 LUSC samples and 30 normal samples in the GSE2088 dataset were analyzed. The threshold of the DEGs between the two groups was set at | log_2_(fold change, FC)|> 1 and *P* value < 0.05. Then, the Kyoto Encyclopedia of Genes and Genomes (KEGG) analysis and Gene Ontology (GO) analysis were used to find out the pathways and the terms in which the common DEGs from these two datasets were enriched. Volcano plots of DEGs, KEGG, and GO analysis from these two datasets were drawn with the R package. The relationship between LSM12 and the overall survival of lung cancer was downloaded and presented with the Kaplan-Meier Plotter database (https://kmplot.com/analysis/).

### Patient samples

Forty-six LUSC tissues and thirty-three normal tissues (bronchial tissues) were collected from the Shengjing Hospital of China Medical University. Prior to surgery, these individuals did not undergo systemic chemotherapy, targeted treatment, or immunotherapy. An informed consent form was signed by each patient. All ethical regulations relevant to human research participants were followed. This study was conducted in accordance with the Declaration of Helsinki’s tenets.

### Cell culture and infection

LUSC cell line NCI-H1703 and NCI-H520 cells were purchased from iCell Bioscience Inc. (Shanghai, China) and cultured in RPMI-1640 medium with 10% fetal bovine serum (FBS) at 37 °C with 5% CO_2_. For overexpression or knockdown of LSM12 in NCI-H1703 and NCI-H520 cells, an LSM12-overexpressing lentiviral vector was constructed using PLJM1-eGFP lentiviral vector, and pLKO.1-eGFP lentiviral vector was inserted with shRNAs targeting LSM12. Subsequently, the lentivirus containing LSM12 overexpression vector (LSM12, short for LSM12-GFP fusion protein), control (Vector), shRNAs targeting LSM12 (^sh1^LSM12, ^sh2^LSM12), and negative control (^sh^NC) were packaged and purified. The LUSC cells were infected with lentivirus containing LSM12, ^sh1^LSM12, or ^sh2^LSM12. The efficiency of infection of lentivirus was observed using GFP fluorescence and Real-time qPCR. The stable LSM12-overexpressing or LSM12-silencing cells were screened and used for further study. The lentivirus-overexpressing SAMD4A, using the same eGFP lentiviral vector (SAMD4A, short for SAMD4A-GFP fusion protein) was infected into LUSC cells or LSM12-overexpressing LUSC cells to study the regulatory mechanism between LSM12 and SAMD4A. Plasmid vectors carrying CDS of ARRB1-L or ARRB1-S were obtained from Youbio (Yongzhou, China) and transfected into LSM12-silencing LUSC cells and incubated 48 h prior to further experiment. In some experiments, cells were treated with 5 μg/ml actinomycin D (Act-D, MCE, USA), an inhibitor of RNA synthesis, for 0, 30, 60, 90, and 120 min, respectively.

### Animals

Male BALB/c nude mice (6 weeks old) were obtained from the Huachuang Sino Pharmatech Co., Ltd (Taizhou, China) and housed at 22 ± 1 °C, 45–55% humidity with a cycle of 12-h light/dark. All mice were given free access to food and water. Experimental cages were placed side by side on the racks. All animal experiments proceeded following the Guide for Care and Use of Laboratory Animals. This study was approved by the Medical Ethics Committee of Shengjing Hospital of China Medical University. We have complied with all relevant ethical regulations for animal use. After a week of adaptive feeding, nude mice were randomly assigned to three groups (*n* = 6 for each group) and suffered from subcutaneous injection on the left and right back with 5×10^6^ NCI-H1703 cells (Vector- or LSM12-overexpressing- cells, ^sh^NC- or ^sh1^LSM12- silencing-cells, ^sh^NC- or ^sh2^LSM12-silencing cells). The length and width of tumors were measured to calculate the tumor size [(length × width^2^)/2]. The mice were euthanized and the tumor tissues of mice were harvested after 30 days of injection, with tumor size less than 17 mm in diameter. The humane endpoint of the nude mouse tumorigenesis experiment was set as when the tumor exceeded 17 mm in diameter, the mice were euthanized to assess exclusion from the experiment. No animals were excluded from this experiment. The images of these tumor tissues were captured. The immunofluorescence staining and immunohistochemistry staining image were photographed by researchers who were blinded to the treatment group.

Male BALB/c nude mice were randomly assigned to five groups (*n* = 6 for each group) and were injected with Vector, LSM12-overexpressing, ^sh^NC, or LSM12-silencing NCI-H1703 cells (1 × 10^6^ cells) through the tail vein. The metastatic lesions were monitored with bioluminescence imaging after 6 weeks of injection. Then, the mice were euthanized to harvest the lung and liver tissues. Images of these tissues were captured, and the numbers of tumor nodules on the lung and liver tissue surfaces were counted.

### Real-time qPCR and reverse transcription-PCR (RT-PCR)

Total RNA was extracted from samples with TRIpure (BioTeke Bio., Beijing, China). The cDNA for Real-time qPCR was synthesized using BeyoRT II M-MLV reverse transcriptase (Beyotime Biotechnology, Shanghai, China). Real-time qPCR proceeded with cDNA, specific primers, and the SYBR green (Solarbio Science & Technology, Co., Ltd., Beijing, China). The primers for Real-time qPCR were listed as: *LSM12*: 5’-TACTTCAGCGTTGGGAGC-3’ (forward) and 5’-GGGTTTCTGTTCGGTCATT-3’ (reverse); *SAMD4A*: 5’-AAAATCCTGGCTCACTCTAT-3’ (forward) and 5’-AAAGCACTACGGTCTTCTAAC-3’ (reverse), *β-actin*: 5’-GGCACCCAGCACAATGAA-3’ (forward) and 5’-TAGAAGCATTTGCGGTGG-3’ (reverse). The levels of mRNA were calculated with a 2^−ΔΔCt^ method. The cDNA for RT-PCR was synthesized, and the PCR amplification was performed, and RT-PCR products were detected by agarose gel electrophoresis. The primers of *ARBB1*-L and *ARBB1*-S for RT-PCR were: 5’-GGGGATCATTGTTTCCTACA-3’ (forward), 5’- CTTCCCGATGCGGGGGTTCC-3’ (reverse).

### Western blot

Total protein extraction was performed using RIPA lysis buffer (Proteintech, Wuhan, China). The concentrations of proteins were measured with a BCA kit (Proteintech). Proteins were separated on sodium dodecyl sulfate-polyacrylamide gel electrophoresis (SDS-PAGE) and transferred onto polyvinylidene fluoride (PVDF) membranes (Thermo Fisher Scientific Inc., Waltham, MA, USA). Then, the membranes were incubated with primary antibodies against LSM12 (1: 20000, Abcam), SAMD4A (1:1000, Proteintech), cleaved caspase-3 (1:500, Affinity Biosciences), E-cadherin (1:1000, ABclonal Technology, Wuhan, China), N-cadherin (1:1000, ABclonal Technology), Flag (1:500, ABclonal Technology), GFP (1:500, ABclonal Technology) and β-actin (1:20000, Proteintech) at 4 °C overnight. PVDF membranes were incubated with secondary antibodies (goat anti-rabbit IgG-HRP, and goat anti-mouse IgG-HRP, Proteintech.) at 37 °C for 40 min. The membranes were exposed to enhanced chemiluminescence (ECL) reagents (Proteintech). The optical density of the target protein was analyzed by a Gel image processing system.

### Cell counting kit-8 (CCK-8)

The proliferation of LUSC cells in different groups was measured using CCK-8 (KeyGen Biotech., Nanjing, China). LUSC cells (5 × 10^3^ cells per well) were inoculated into 96-well plates. Cells were cultured for 0, 24, 48, and 72 h at 37 °C with 5% CO_2_. Then, cells were treated with ten μl CCK-8/well at 37 °C for 2 h. The optical density (OD) at 450 nm was examined with a microplate reader (BioTek Instruments, Winooski, VT, USA).

### Immunofluorescence assay

The expressions of Ki67 and E-cadherin in LUSC cells were visualized with an immunofluorescence assay, respectively. Each cell coverslip was fixed in 4% paraformaldehyde (Sinopharm Chemical Reagent Co., Ltd., Shanghai, China) for 15 min. Then, cells were incubated with 0.1% tritonX-100 (Beyotime Biotechnology) for 30 min to enhance the permeability of the cell membrane. 1% Bovine Serum Albumin (BSA, Sangon Biotech, Shanghai, China) was added into cells to block nonspecific sites for 15 min. Then, cells were incubated with Ki67 (1:100, Affinity Biosciences) or E-cadherin (1:100, ABclonal Technology) antibodies at 4 °C overnight and then incubated with the secondary antibody in the dark for 60 min. Cell nuclei were stained with DAPI (Aladdin, Shanghai, China). The expression of Ki67 in tumor tissues was detected. The tumor tissues were dehydrated in a gradient of alcohol and infiltrated with xylene. Then, the tissues were embedded in paraffin and sliced into 5-μm sections. The sections were subjected to an immunofluorescence assay using an anti-Ki67 antibody. The images of immunofluorescence staining were captured with a microscope.

### TdT-mediated dUTP Nick-End Labeling (TUNEL) staining

Cell coverslips were incubated with 200 μl 0.1% tritonX-100 for 15 min. Then, cells were incubated in TUNEL staining solution (Roche, Basel, Switzerland) in the dark at 37 °C for 60 min. After PBS washing, the nuclei were stained with DAPI for 5 min, and the positive staining cell number and total cell number were counted, and the rate of apoptotic cells was calculated using the formula: (Number of TUNEL staining cells)/(Number of total cells) × 100%.

### Flow cytometry (FCM)

The cell cycle was detected using a Cell cycle kit (KeyGen Biotech). Briefly, LUSC cells were centrifuged at 150 *g* for 5 min and incubated in pre-cooled 70% ethanol at 4 °C overnight. The cells were centrifuged and resuspended with 500 μl PI/RNase A staining solution. After incubating in the dark for 30 min, cell cycle was detected using FCM.

### Caspase-3 activity assay

The activity of caspase-3 was measured with the Caspase-3 Activity Assay Kit (Beyotime Biotechnology). Briefly, the protein concentration of cells was assessed with the Bradford Protein Assay Kit (Beyotime Biotechnology). Then, the caspase-3 substrate pNA standard curve was measured. Samples were incubated with Ac-DEVD-pNA at 37 °C for 2 h. Finally, the content of pNA, which was produced from samples, was calculated according to the pNA standard curve.

### Transwell assays

Cells were suspended in a serum-free medium. In the cell migration assay, the 200 μl cell suspension was placed into the top chamber of the transwell with a serum-free medium, and the 800 μl medium with 10% FBS was added into the bottom chamber. In the cell invasion assay, Matrigel (Corning, NY, USA) was added to the membrane of the transwell insert and incubated for two hours to solidify the Matrigel. The cells were added to the top chamber of the transwell. After 24 h, cells from the bottom chamber were washed with PBS, fixed with 4% paraformaldehyde, and then stained with 0.5% crystal violet (Amresco, Solon, OH, USA) for 5 min. The numbers of cells were counted using a microscope.

### Immunohistochemical (IHC) analysis

LSM12 expressions in tumor tissues from clinical patients and mice were visualized with IHC staining. Sections were incubated with 3% H_2_O_2_ for 15 min to inactivate the endogenous peroxidases. Then, sections were blocked with 1% BSA for 15 min and incubated with an anti-LSM12 antibody (1:50, Abcam) at 4 °C overnight and incubated with a second antibody in the dark for 45 min. The sections were visualized with a DAB solution (Sangon Biotech), followed by staining with hematoxylin (Solarbio Science & Technology, Co., Ltd.). Images were captured with a microscope. The percentage of positive cells (0–100%) was multiplied by the intensity (0–3) to determine the H-score for IHC staining, which produced a continuous value between 0 and 300^39^. The median of the H-score was then used as a cutoff value to divide the H-scores into low and high LSM12 expression, and they were subsequently correlated with clinicopathological features.

### RNA immunoprecipitation (RIP)-PCR

RIP assay was performed by EZ-Magna RIP Kit (Millipore, Billerica, MA, USA) following the instructions. In brief, cells were lysed with RIP lysis buffer. Magnetic beads were resuspended in RIP washing buffer and cultured with antibodies. Then, complexes of magnetic beads and the anti-SAMD4A (Proteintech) or anti-LSM12 antibody (Abcam) were mixed and rotated for 30 min, followed by adding the post-centrifuged supernatant of cell lysates and rotating at 4 °C for at least 3 h. Subsequently, the RNA-protein complexes were washed, and the precipitated RNAs were purified. Then, the target RNA was detected by RT-PCR. The RIP-PCR primers of LSM12 were: LSM12, 5’-AGCCAATGAGCAGAAAAC-3’ (forward), 5’-AAAAGGAAGGGGAAAGAG-3’ (reverse). The primers of IL1β were: 5’- ACAGTGGCAATGAGGATGACTTGTTCT-3’ (forward), 5’- TCAGGTCATTCTCCTGGAAGGTCTG-3’ (reverse). The primers of THBS1 were: 5’-ACAGTTCCTGATGGAGAATGCTGTCCT-3’ (forward), 5’- CACCATCCTGTTTAAATCTCTTGTCACA-3’ (reverse). The primers of ARBB1 were 5’-CGTACAGTCGTTCCCACC-3’ (forward) and 5’-GGATGACCAGACGCACAG-3’ (reverse). The products of RT-PCR were analyzed with agarose gel electrophoresis.

### mRNA-seq and alternative splicing (AS) analysis

The mRNA-seq assay was performed using LSM12-overexpressing and negative control NCI-H1703 cells to detect the DEGs that LSM12 regulates. RNA-seq was performed by Novogene Corporation (Beijing, China). The Ultra RNA Library Prep Kit for Illumina (NEB, USA) constructed the mRNA library. The threshold of the DEGs was set at | log_2_FC|> 1 and *P* value < 0.05. Principal component analysis (PCA) plot for quality control of samples and the heatmap for the unsupervised hierarchal clustering of DEGs were drawn using the R package.

The AS events were analyzed by the rMATS software^40^. The mRNA-seq data of NCI-H1703 cells with LSM12 overexpression or control vector (*N* = 3) were used for AS analysis. AS events were classified into SE (skipped exon), MXE (mutually exclusive exon), A5SS (alternative 5′ splice site), A3SS (alternative 3′ splice site), and RI (retained intron). The AS events were quantified using inclusion level 1 (IncLevel1) from the vector group and inclusion level 2 (IncLevel2) from the LSM12-overexpressing group. Differential AS events were considered if they passed the following threshold: | IncLevelDifference = (IncLevel1- IncLevel2)|> 0.1 and *p* < 0.05.

### RIP-seq assay

LSM12 RIP-seq was used to identify binding targets of LSM12 and was conducted by Novogene Corporation. A library was constructed and then sequenced using the Illumina platform following the manufacturer’s recommendations. The motif analysis was conducted using HOMER. Peak-related genes were confirmed by Peak Annotator. Subsequently, the GO and KEGG enrichment analysis of the overlapped genes, including the DEGs from mRNA-seq and identified genes from RIP-seq, was performed.

### RNA pull-down assay

RNA pull-down assay was performed with the Pierce™ Magnetic RNA-Protein Pull-Down Kit (Thermo Fisher Scientific Inc.). Briefly, cells were lysed with IP Lysis Buffer. LSM12 RNA probe (AAUGAUUGAAAGAAACUUUUACAUCUU) containing 520–534 of LSM12 3’ UTR and IL1β-probe (AUUCUGAUGAGCAACCGCUUCCCUAUU)^18^ were synthesized by JTS (Wuhan, China). A scrambled probe (non-sense sequence) as a negative control was purchased from Geneseed Biotech (Guangzhou, China). Then, magnetic beads were washed with 20 mM Tris and were incubated with lysate at 4 °C for 30–60 min to precipitate the RNA-protein complexes. The RNA-protein complexes were washed with wash buffer, and the proteins were eluted from the magnetic beads and subjected to western blot analysis.

### Dual luciferase reporting assay

HEK293T cells were purchased from iCell Bioscience Inc. and cultured in a DMEM medium with 10% FBS. SAMD4A was overexpressed by inserting the CDS of SAMD4A into the pcDNA3.1 plasmid vector. The plasmid vector was as a negative control (NC). To explore the role of SAMD4A on the stability of *LSM12* mRNA, HEK293T cells were co-transfected with SAMD4A overexpressed plasmid and the pmirGLO luciferase reporter harboring the sequence of wild-type (WT) LSM12 3’ UTR region or mutant (MUT) LSM12 3’ UTR through replacing two nucleotides. Luciferase activity was measured with a dual-luciferase reporter gene assay kit (KeyGen Biotech) according to the manufacturer’s suggestion, and the renilla luciferase was used as an internal control.

### Statistics and reproducibility

Data analysis was performed using GraphPad Prism (GraphPad Software, CA, USA). Unpaired Student’s t-test was used to analyze the differences between the two groups. Correlations between LSM12 expression and clinicopathological features were analyzed by the Chi-square test. Analysis of variance (ANOVA) was used to analyze the differences among multiple groups. All experiments were performed at least three times, and with results were presented as mean ± standard deviation (SD), and the significance threshold was set at *p* < 0.05.

### Reporting summary

Further information on research design is available in the Nature Portfolio Reporting Summary linked to this article.

## Supplementary information

Supplementary Information

Description of Additional Supplementary Files

Supplementary data 1

Supplementary data 2

Supplementary data 3

Supplementary data 4

Reporting Summary

## Data Availability

The RNA-seq datasets of LUSC and normal lung tissues are downloaded from GEO database (GSE2088, https://www.ncbi.nlm.nih.gov/gds/) and the GEPIA database (http://gepia.cancer-pku.cn/). The relationship between LSM12 and the overall survival of lung cancer was obtained from the Kaplan-Meier Plotter database (https://kmplot.com/analysis/). The expression levels of LSM12 in cancer stages of LUSC were obtained UALCAN database (https://ualcan.path.uab.edu/index.html). Our RNA-seq data of LSM12 overexpressing cells have been deposited at GEO (GSE295673), and data analyzed during this study, and source data in figures are presented in Supplementary Data 1-4. Uncropped/unedited blots are provided in the Supplementary information as Supplementary Fig. 4.
