# Supplementary material for: LSM12 promotes the lung squamous cell carcinoma progression through mediating alternative splicing of ARRB1
Source: Commun Biol. 2025 May 27;8:814. doi: 10.1038/s42003-025-08193-7 (PMC12116798; doi:10.1038/s42003-025-08193-7)

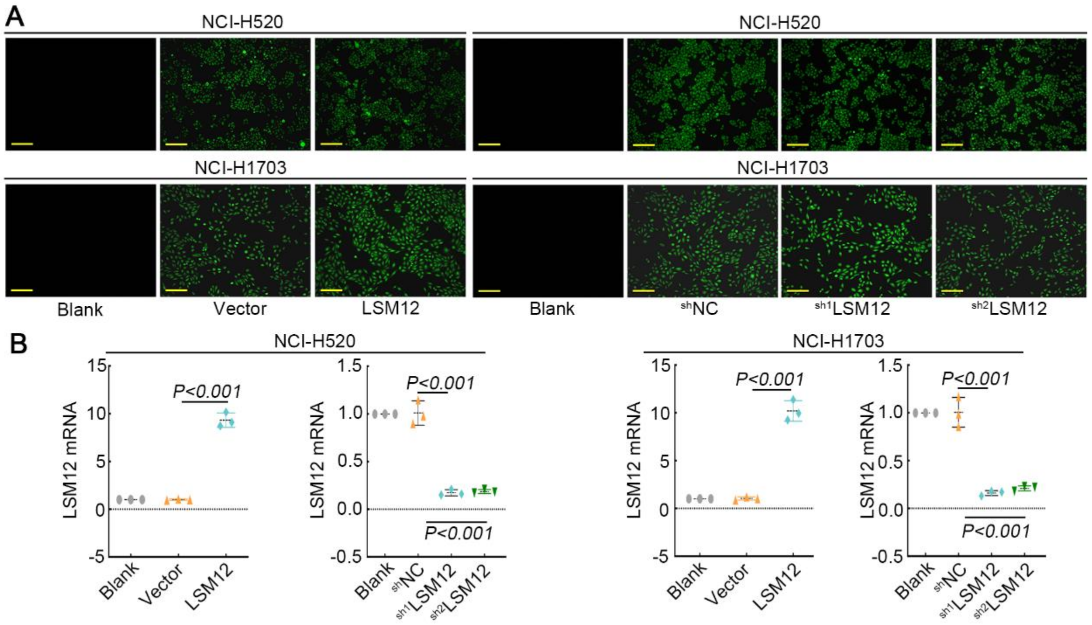

Supplementary Figure 1. LSM12 expression in LSM12-overexpressing or LSM12-silencing cells.

A Fluorescence of GFP of LUSC cells after infecting with lentivirus to overexpress or silence LSM12. The scale bar was 200 μm. **B** The mRNA of LSM12 was detected by real-time qPCR. N = 3, and the data were presented as mean ± standard deviation (SD). Significant differences were indicated as $p<0.05$.

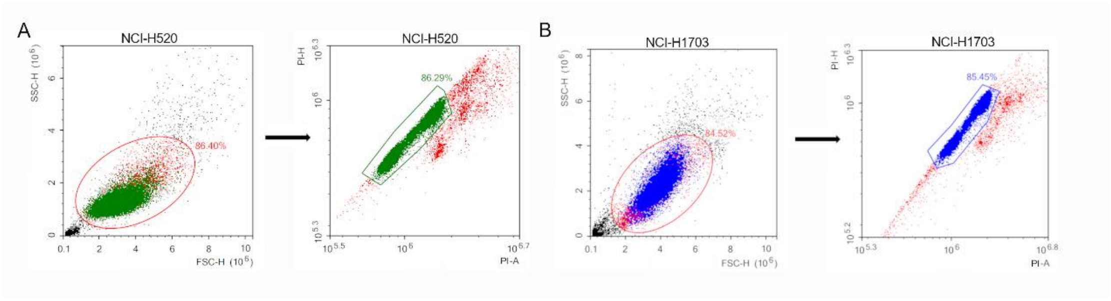

Supplementary Figure 2. Representative strategies for flow cytometry analysis of NCI-H520 and NCI-1703 cells

Representative strategies for flow cytometry analysis of NCI-H520 (**A**) and NCI-1703 (**B**) cells.

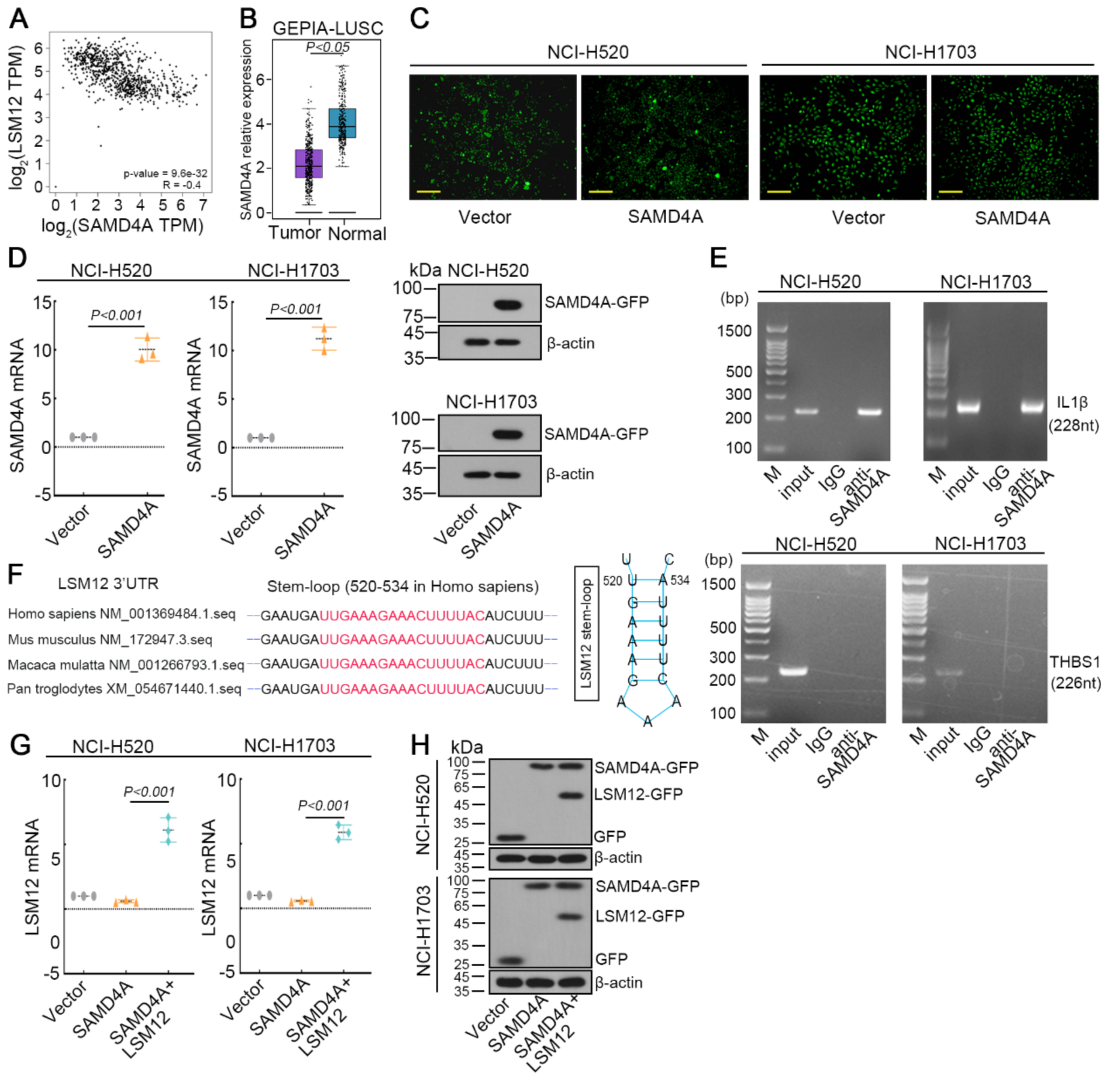

Supplementary Figure 3. **LSM12 is a downstream of SAMD4A.**

A The correlation analysis between SAMD4A and LSM12 mRNA expression was obtained from the GEPIA database. **B** The mRNA expression of SAMD4A in the GEPIA dataset. **C** LUSC cells were infected with lentivirus to overexpress SAMD4A and the SAMD4A expression were detected using GFP fluorescence. The scale bar was 200 µm. **D** The mRNA expression of SAMD4A in LUSC cells was measured with Real-time qPCR. The protein levels of SAMD4A were detected using an anti-GFP antibody by western blot. **E** The interactions between SAMD4A and IL1β (positive target control), or SAMD4A and THBS1 (negative control) were detected with RIP-PCR. **F** The conserved LSM12 3'UTR sequences from different species. The stem loop sequence was predicted by ViennaRNA Web Services. **G** The mRNA expression of LSM12 after overexpressing SAMD4A and LSM12 was detected by Real-time qPCR. **H** The protein levels of SAMD4A and LSM12 were detected using an anti-GFP antibody. N = 3, and the data were presented as mean ± standard deviation (SD). Significant differences were indicated as $p<0.05$.

Supplementary Figure 4. All original immunoblot images.

Figure 4B.

LSM12 overexpression in NCI-H520 cells

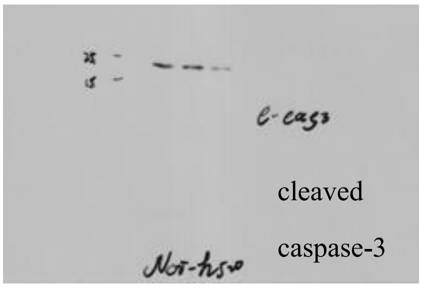

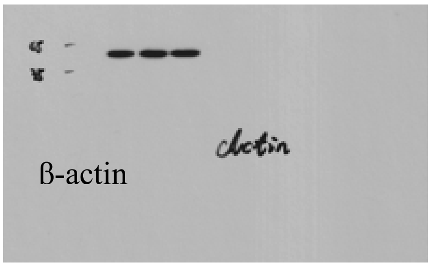

LSM12 knockdown in NCI-H520 cells

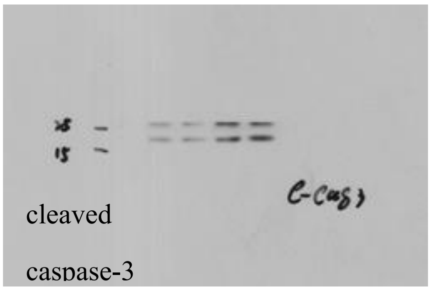

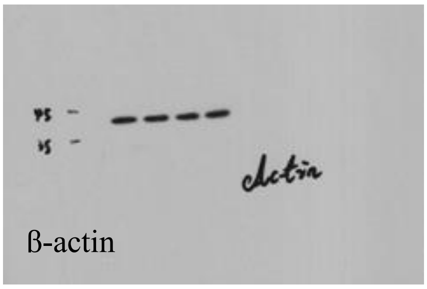

LSM12 overexpression in NCI-H1703 cells

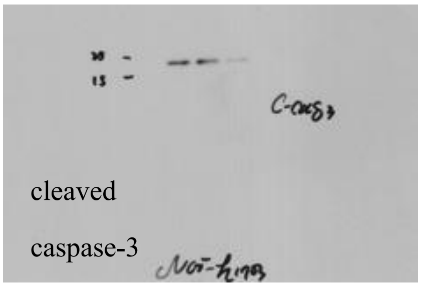

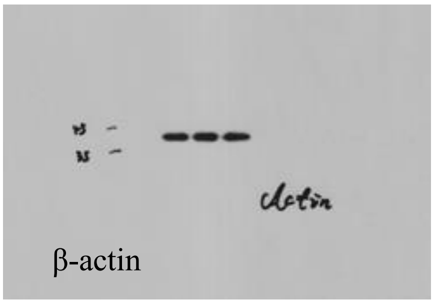

LSM12 knockdown in NCI-H1703 cells

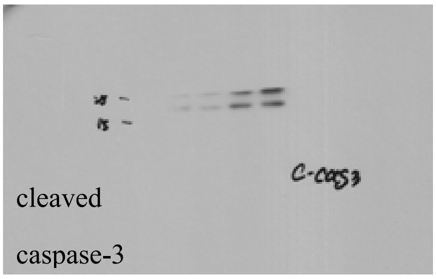

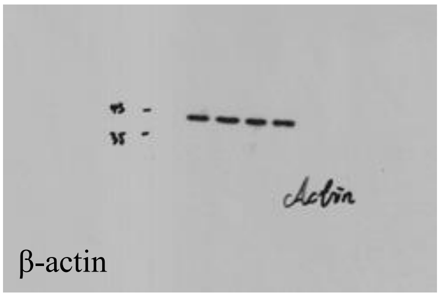

Figure 5C.

LSM12 knockdown in NCI-H520 cells

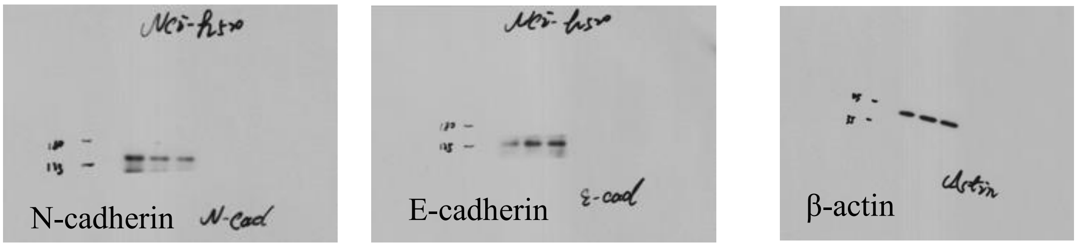

LSM12 overexpression in NCI-H520 cells

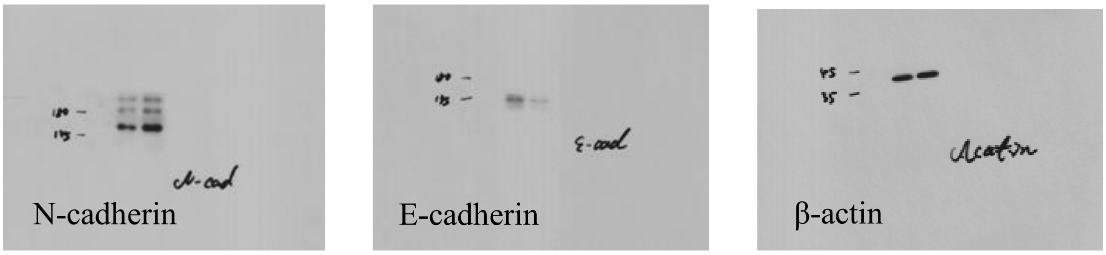

LSM12 knockdown in NCI-H1703 cells

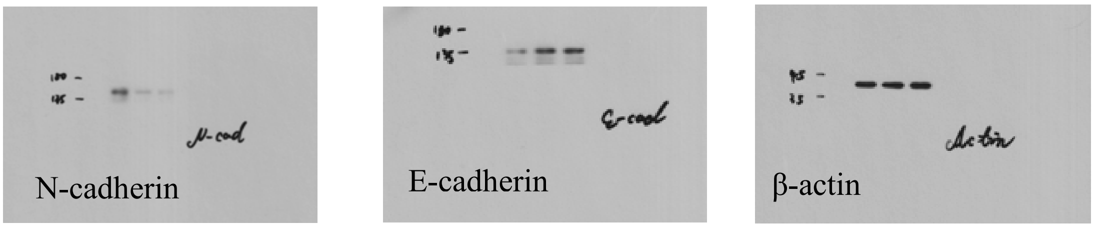

LSM12 overexpression in NCI-H1703 cells

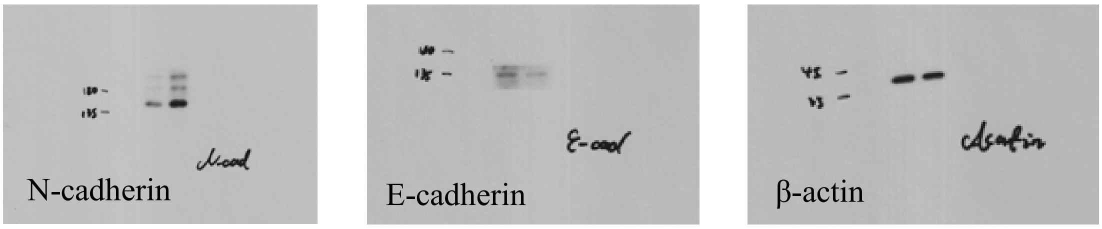

Figure 7B.

SAMD4A overexpression in NCI-H520 cells

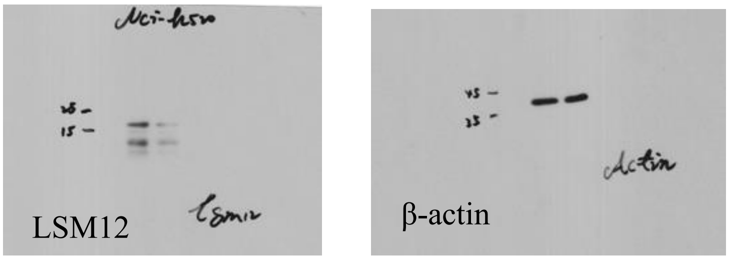

SAMD4A overexpression in NCI-H1703 cells

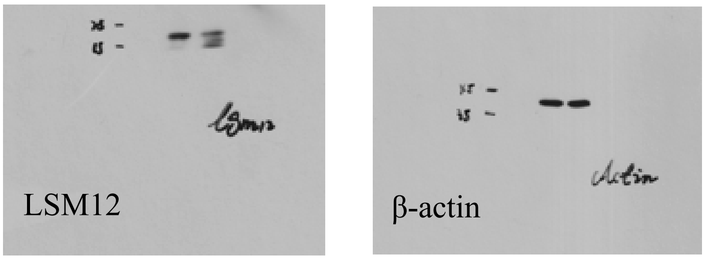

Figure 7E. RNA pull-down

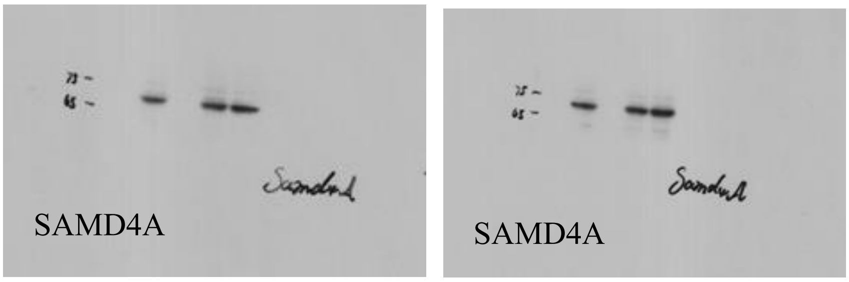

Figure 7I.

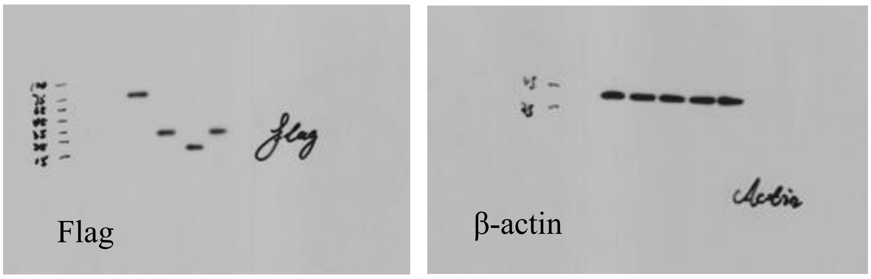

Figure 7M.

SAMD4A and LSM12 overexpression in NCI-H520 cells and NCI-H1703 cells

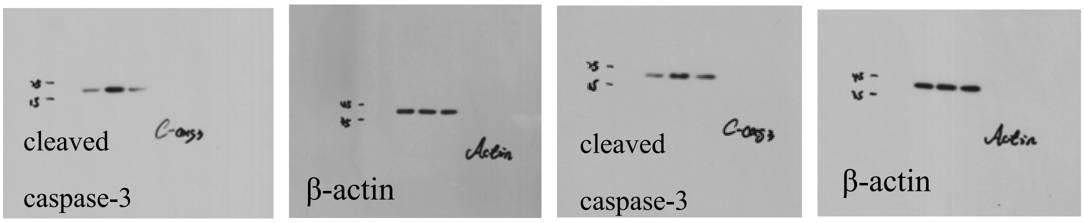

Figure S2D.

SAMD4A overexpression in NCI-H520 cells

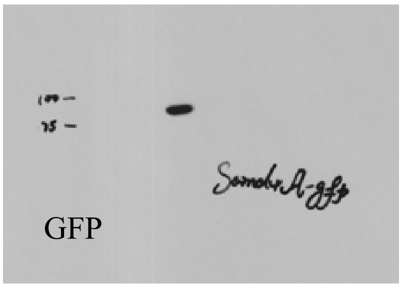

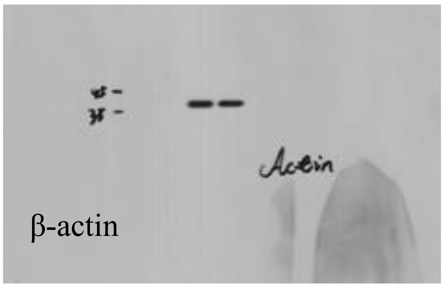

SAMD4A overexpression in NCI-H1703 cells

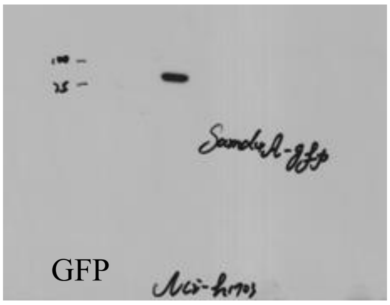

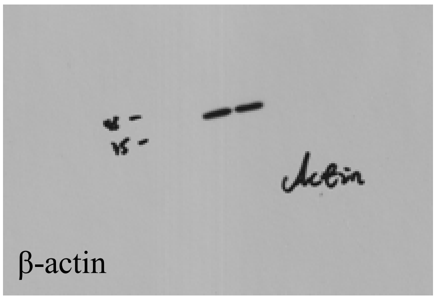

Figure S2H.

SAMD4A and LSM12 overexpression in NCI-H520 cells

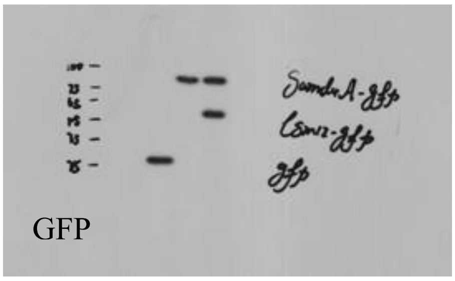

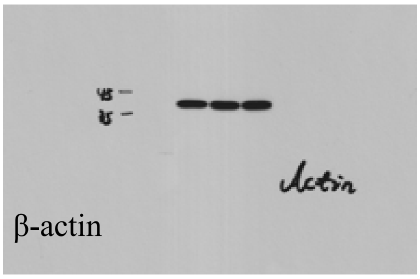

SAMD4A and LSM12 overexpression in NCI-H1703 cells

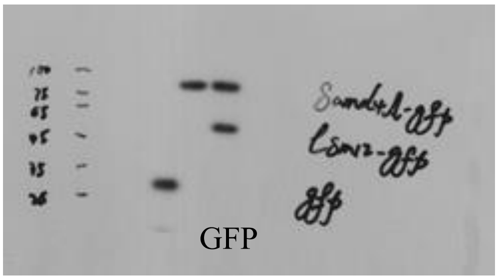

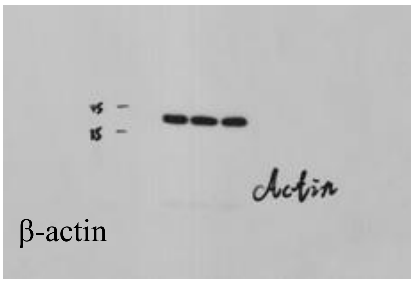

Supplement: Supplementary file 1 — Supplementary Information [file 42003_2025_8193_MOESM1_ESM.pdf]
