# Supplementary material for: LSM12 promotes the lung squamous cell carcinoma progression through mediating alternative splicing of ARRB1
Source: Commun Biol. 2025 May 27;8:814. doi: 10.1038/s42003-025-08193-7 (PMC12116798; doi:10.1038/s42003-025-08193-7)
Supplement: Supplementary file 2 — Description of Additional Supplementary Files [file 42003_2025_8193_MOESM2_ESM.docx]

Description of Additional Supplementary Files

**File name:** Supplementary Data 1

**Description:** The expressed genes from mRNA-seq for Figure 8 and 9.

**File name:** Supplementary Data 2

**Description:** The identified targets of LSM12 from RIP-seq for Figure 8 and 9.

**File name:** Supplementary Data 3

**Description:** The data of alternative splicing (AS) events for Figure 9.

**File name:** Supplementary Data 4

**Description:** The source data for the graphs in Figures in the paper.
