# Supplementary material for: LSM12 promotes the lung squamous cell carcinoma progression through mediating alternative splicing of ARRB1
Source: Commun Biol. 2025 May 27;8:814. doi: 10.1038/s42003-025-08193-7 (PMC12116798; doi:10.1038/s42003-025-08193-7)
Supplement: Supplementary file 7 — Reporting Summary [file 42003_2025_8193_MOESM7_ESM.pdf]

nature portfolio

Corresponding author(s): Gang Zhao
Huanhuan Chen

Last updated by author(s): Apr 29, 2025

Reporting Summary

Nature Portfolio wishes to improve the reproducibility of the work that we publish. This form provides structure for consistency and transparency in reporting. For further information on Nature Portfolio policies, see our Editorial Policies and the Editorial Policy Checklist.

Statistics

For all statistical analyses, confirm that the following items are present in the figure legend, table legend, main text, or Methods section.

n/a	Confirmed	
☐	☒	The exact sample size (*n*) for each experimental group/condition, given as a discrete number and unit of measurement
☐	☒	A statement on whether measurements were taken from distinct samples or whether the same sample was measured repeatedly
☐	☒	The statistical test(s) used AND whether they are one- or two-sided<br>*Only common tests should be described solely by name; describe more complex techniques in the Methods section.*
☒	☐	A description of all covariates tested
☐	☒	A description of any assumptions or corrections, such as tests of normality and adjustment for multiple comparisons
☐	☒	A full description of the statistical parameters including central tendency (e.g. means) or other basic estimates (e.g. regression coefficient) AND variation (e.g. standard deviation) or associated estimates of uncertainty (e.g. confidence intervals)
☐	☒	For null hypothesis testing, the test statistic (e.g. *F*, *t*, *r*) with confidence intervals, effect sizes, degrees of freedom and *P* value noted<br>*Give P values as exact values whenever suitable.*
☒	☐	For Bayesian analysis, information on the choice of priors and Markov chain Monte Carlo settings
☒	☐	For hierarchical and complex designs, identification of the appropriate level for tests and full reporting of outcomes
☒	☐	Estimates of effect sizes (e.g. Cohen's *d*, Pearson's *r*), indicating how they were calculated

Our web collection on statistics for biologists contains articles on many of the points above.

Software and code

Policy information about availability of computer code

Data collection	Data analysis was performed using GraphPad Prism (GraphPad Software, Ca, USA).
Data analysis	Data analysis was performed using GraphPad Prism (GraphPad Software, Ca, USA). Unpaired Student's t-test was used to analyze the differences between the two groups. Correlations between LSM12 expression and clinicopathological features were analyzed by chi-square test. Analysis of variance (ANOVA) was used to analyze the differences among multiple groups. All experiments were performed at least three times, with results were presented as mean ± standard deviation (SD), and the significance threshold was set at p <0.05.

For manuscripts utilizing custom algorithms or software that are central to the research but not yet described in published literature, software must be made available to editors and reviewers. We strongly encourage code deposition in a community repository (e.g. GitHub). See the Nature Portfolio guidelines for submitting code & software for further information.

Data

Policy information about availability of data

All manuscripts must include a data availability statement. This statement should provide the following information, where applicable:

- Accession codes, unique identifiers, or web links for publicly available datasets
- A description of any restrictions on data availability
- For clinical datasets or third party data, please ensure that the statement adheres to our policy

The RNA-seq datasets are accessible through GEO series accession number GSE2088 (https://www.ncbi.nlm.nih.gov/gds/) and GEPIA database (http://gepia.cancer-

pku.cn/). The relationship between LSM12 and the overall survival of lung cancer was obtained from Kaplan-Meier Plotter database (https://kmplot.com/analysis/). The expression levels of LSM12 in cancer stages of LUSC were obtained UALCAN database (https://ualcan.path.uab.edu/index.html). RNA-seq data has been deposited at GEO (GSE295673) and data analyzed during this study and source data in figures are presented in Supplementary Data 1-4. Uncropped/unedited blots are provided in the Supplementary information as Supplementary Figure 4.

Human research participants

Policy information about studies involving human research participants and Sex and Gender in Research.

Reporting on sex and gender	The gender of the sample was chosen randomly. Because it was a lung cancer study, there were more male patients.
Population characteristics	The patients did not undergo systemic chemotherapy, targeted treatment, or immunotherapy. Because one of the important causes of lung cancer is smoking, lung cancer patients are mostly male.
Recruitment	Forty-six LUSC tissues and thirty-three normal tissues from LUSC patients were collected.
Ethics oversight	This study was approved by the Medical Ethics Committee of Shengjing Hospital of China Medical University.

Note that full information on the approval of the study protocol must also be provided in the manuscript.

Field-specific reporting

Please select the one below that is the best fit for your research. If you are not sure, read the appropriate sections before making your selection.

☒ Life sciences ☐ Behavioural & social sciences ☐ Ecological, evolutionary & environmental sciences

For a reference copy of the document with all sections, see nature.com/documents/nr-reporting-summary-flat.pdf

Life sciences study design

All studies must disclose on these points even when the disclosure is negative.

Sample size	mice experiments: sample size = 6; cell experiments: sample size = 3.
Data exclusions	No data were excluded.
Replication	All experiments were performed at least three times of biological duplication.
Randomization	The experimental animal and cells were randomly assigned to different groups before any treatment.
Blinding	The immunofluorescence staining and immunohistochemistry staining image were photographed by researchers who were blinded to the treatment group.

Reporting for specific materials, systems and methods

We require information from authors about some types of materials, experimental systems and methods used in many studies. Here, indicate whether each material, system or method listed is relevant to your study. If you are not sure if a list item applies to your research, read the appropriate section before selecting a response.

Materials & experimental systems

n/a	Involved in the study	
☐	☒	Antibodies
☐	☒	Eukaryotic cell lines
☒	☐	Palaeontology and archaeology
☐	☒	Animals and other organisms
☐	☒	Clinical data
☒	☐	Dual use research of concern

Methods

n/a	Involved in the study	
☒	☐	ChIP-seq
☐	☒	Flow cytometry
☒	☐	MRI-based neuroimaging

Antibodies

Antibodies used	LSM12 (ab173292, Abcam); SAMD4A (17387-1-AP, Proteintech); cleaved caspase-3 (AF7022, Affinity); E-cadherin (A20798, ABclonal

Antibodies used	Technology), N-cadherin (A19083, ABclonal Technology); Flag (AE063, ABclonal Technology),GFP (AE011, ABclonal Technology), and β-actin (66009-1-Ig, Proteintech); Ki67 (AF0198, Affinity).
Validation	All antibodies are commercially available have been tested for species reactivity by the manufactures.

Eukaryotic cell lines

Policy information about cell lines and Sex and Gender in Research

Cell line source(s)	LUSC cell line NCI-H1703 and NCI-H520 cells were purchased from iCell Bioscience Inc (Shanghai, China).
Authentication	STR authentication from the manufacturers.
Mycoplasma contamination	All cell lines tested negative for mycoplasma contamination.
Commonly misidentified lines (See ICLAC register)	No commonly misidentified lines.

Animals and other research organisms

Policy information about studies involving animals; ARRIVE guidelines recommended for reporting animal research, and Sex and Gender in Research

Laboratory animals	BALB/c nude mice.
Wild animals	N/A
Reporting on sex	Male.
Field-collected samples	N/A
Ethics oversight	The experimental procedures were approved by the Medical Ethics Committee of Shengjing Hospital of China Medical University.

Note that full information on the approval of the study protocol must also be provided in the manuscript.

Clinical data

Policy information about clinical studies

All manuscripts should comply with the ICMJE guidelines for publication of clinical research and a completed CONSORT checklist must be included with all submissions.

Clinical trial registration	Not a Clinical trial registration. The expression of LSM12 from the pathological sections of tumor and normal tissues was detected using IHC staining assay.
Study protocol	*Note where the full trial protocol can be accessed OR if not available, explain why.*
Data collection	*Describe the settings and locales of data collection, noting the time periods of recruitment and data collection.*
Outcomes	*Describe how you pre-defined primary and secondary outcome measures and how you assessed these measures.*

Flow Cytometry

Plots

Confirm that:

☒ The axis labels state the marker and fluorochrome used (e.g. CD4-FITC).

☒ The axis scales are clearly visible. Include numbers along axes only for bottom left plot of group (a 'group' is an analysis of identical markers).

☒ All plots are contour plots with outliers or pseudocolor plots.

☒ A numerical value for number of cells or percentage (with statistics) is provided.

Methodology

Sample preparation	LUSC cells were centrifuged at 150 g for 5 min and incubated in precooled 70% ethanol at 4 °C overnight. The cells were centrifuged and resuspended with 500 μl PI/RNase A staining solution. After incubating in the dark for 30 min, cell cycle was detected using FCM.
Instrument	flow cytometer (NovoCyte, Agilent)

Software	NovoExpress
Cell population abundance	The abundance of the LUSC cell percentage in the G0/G1 phase, S phase and G2/M phase.
Gating strategy	Gating most of cells in the preliminary FSC/SSC gates. No staining cells were used for a negative control.

☒ Tick this box to confirm that a figure exemplifying the gating strategy is provided in the Supplementary Information.
